# Metagenome-validated combined amplicon sequencing and text mining-based annotations for simultaneous profiling of bacteria and fungi: vaginal microbiota and mycobiota in healthy women

**DOI:** 10.1186/s40168-024-01993-9

**Published:** 2024-12-28

**Authors:** Seppo Virtanen, Schahzad Saqib, Tinja Kanerva, Rebecka Ventin-Holmberg, Pekka Nieminen, Tiina Holster, Ilkka Kalliala, Anne Salonen

**Affiliations:** 1 Department of Obstetrics and Gynaecology, University of Helsinki and Helsinki University Hospital, Helsinki, Finland; 2 Faculty of Medicine, Human Microbiome Research Program, University of Helsinki, Helsinki, Finland; 3 Folkhälsan Research Center, 00250 Helsinki, Finland; 4 Department of Surgery and Cancer, Faculty of Medicine, Imperial College London, London, UK; 5 Present Address: Research and Development, Kemira Oyj, Helsinki, Finland

**Keywords:** Microbiota, Mycobiota, Parallel sequencing, 16S rRNA gene, ITS, Text mining, Metagenomics, Vagina

## Abstract

**Background:**

Amplicon sequencing of kingdom-specific tags such as 16S rRNA gene for bacteria and internal transcribed spacer (ITS) region for fungi are widely used for investigating microbial communities. So far most human studies have focused on bacteria while studies on host-associated fungi in health and disease have only recently started to accumulate. To enable cost-effective parallel analysis of bacterial and fungal communities in human and environmental samples, we developed a method where 16S rRNA gene and ITS1 amplicons were pooled together for a single Illumina MiSeq or HiSeq run and analysed after primer-based segregation. Taxonomic assignments were performed with Blast in combination with an iterative text-extraction-based filtration approach, which uses extensive literature records from public databases to select the most probable hits that were further validated by shotgun metagenomic sequencing.

**Results:**

Using 50 vaginal samples, we show that the combined run provides comparable results on bacterial composition and diversity to conventional 16S rRNA gene amplicon sequencing. The text-extraction-based taxonomic assignment-guided tool provided ecosystem-specific bacterial annotations that were confirmed by shotgun metagenomic sequencing (VIRGO, MetaPhlAn, Kraken2). Fungi were identified in 39/50 samples with ITS sequencing while in the metagenome data fungi largely remained undetected due to their low abundance and database issues. Co-abundance analysis of bacteria and fungi did not show strong between-kingdom correlations within the vaginal ecosystem of healthy women.

**Conclusion:**

Combined amplicon sequencing for bacteria and fungi provides a simple and cost-effective method for simultaneous analysis of microbiota and mycobiota within the same samples. Conventional metagenomic sequencing does not provide sufficient fungal genome coverage for their reliable detection in vaginal samples. Text extraction-based annotation tool facilitates ecosystem-specific characterization and interpretation of microbial communities by coupling sequence homology to microbe metadata readily available through public databases.

Video Abstract

**Supplementary Information:**

The online version contains supplementary material available at 10.1186/s40168-024-01993-9.

## Introduction

Profiling of bacterial communities using 16S rRNA gene amplicon sequencing is the standard tool for analysing the composition and diversity of microbiota in human and environmental samples. The fungal component of the microbiota, also called mycobiota, has a long history of research in mycorrhizal fungi and other environmental samples but has only recently gained interest as part of human commensal microbiota. Based on recent molecular studies, there are an estimated 10^12^–10^13^ fungi compared to 10^13^–10^14^ bacteria in the human microbiota, across the gastrointestinal tract, oral cavity, vaginal mucosa, and skin [1]. The most abundant fungi colonizing humans belong to phyla Ascomycota and Basidiomycota. The common fungal genera found in the human mouth, gut, skin, lungs, and vagina in healthy individuals as well as in selected diseases have been extensively reviewed elsewhere [1, 2]. Fungi can interact with the host as well as the rest of the microbiota in both mutualistic and antagonistic ways, highlighting their relevance for human health [3, 4, 5, 6].

For the vaginal ecosystem, the bacterial composition is well described. For most healthy women, it typically consists of one or two *Lactobacillus* species, namely *L. crispatus, L. iners, L. gasseri,* or *L. jensenii,* while the presence of *Gardnerella vaginalis* and an assortment of other, typically anaerobic species is indicative of dysbiosis and prevail, e.g. in bacterial vaginosis (BV) [7]. Different lactobacilli have distinct but only partially characterized roles in the vaginal ecosystem, with *L. crispatus* being the most protective for health and *L. iners* the least [8, 9]. The *Lactobacillus* species detected in the human vagina are genomically and functionally distinct [8, 10, 11] and their abundance and prevalence are associated with various female health outcomes and characterise the well-established vaginal community state types (CSTs) [8, 10, 12]. Hence, species-level identification is a mandatory attempt to study the bacterial microbiota of this ecosystem.

The fungal component of the vaginal ecosystem is poorly characterized, as after the pioneering research of Drell et al. in 2013 [13] where they characterized the vaginal mycobiota of 251 and microbiota of 494 women, progress has been slow. Only a handful of original papers have been published on the vaginal mycobiota, yet it has been associated with various diseases, most obviously yeast infections, but also with vulvodynia, bacterial dysbiosis, and cervical dysplasia [14, 15, 16]. Despite the clear relevance of fungi and interest in cross-kingdom studies, there are limited tools available for such analyses. While metagenomics, i.e. whole genome shotgun sequencing (WGS) in principle allows sequencing of entire microbial communities, in practice the low relative abundance of fungi (e.g. in the vagina in the range of 0.17 ± 0.04%, if detected at all [17] and in the gut 0.01% [18]), is a hindrance for a cost-effective analysis. For over 20 years, the internal transcribed spacer (ITS) region of fungi has been used for their molecular analysis. Currently, there is a rapidly growing number of studies that have processed and sequenced both 16S rRNA gene and ITS amplicons separately for parallel characterization of fungal and bacterial communities in the same samples. In biomedical research, these include studies addressing the impact of sample storage and DNA extraction [19, 20], descriptive compositional analyses of human samples in cross-sectional [13] and longitudinal settings [14, 21], and their correlation to (disease) phenotypes [22, 23, 24, 25]. To our knowledge, there were no other similar studies present at the time of our initial submission in 2021 (https://doi.org/10.21203/rs.3.rs-321778/v1.), however, during the review process of our resubmission in 2023, another study was published that utilised a similar methodology to simultaneously obtain both bacterial and fungal profiles from oral samples [26].

Investigating the microbiota of a taxonomically homogenous community, such as the vaginal microbiota consisting of one or few species of functionally distinct *Lactobacillus*, makes it necessary to seek species-level taxonomic annotations. At present, most amplicon sequencing workflows employ sequence homology as the primary criterion for taxonomic classification and make use of curated databases to improve performance and accuracy such as SILVA [27], RDP [28], GreenGenes [29] and UNITE [30]. However, distinguishing closely related taxa at the species level can be impossible or unreliable with limited amplicon sizes especially when using sequence homology alone. While curated databases are an excellent resource, they tend to be incomplete or prone to mis-annotations [31, 32]. Alternatively, the BLAST alignment tool is extensively used alongside the NCBI nucleotide (nt) and/or the curated 16S rRNA gene/ITS databases for taxonomic annotations. The resulting annotation hits are coupled with extensive sequence alignment metrics such as ‘bitscore,’ ‘percentage identity,’ and ‘query coverage,’ which may be identical between multiple hits representing several distinct taxa. In most cases, extensive post-alignment curation is necessary to select the best hits based on known ecological and biological information.

To address the need for affordable and robust methods to also study the fungi of the human microbiota alongside bacteria, we developed a parallel amplicon sequencing technique that combines the bacterial 16S rRNA gene and the fungal ITS1 region in a single Illumina sequencing run. Here, we study the feasibility and performance of this method by applying it to vaginal samples from 50 healthy women and characterizing their bacterial and fungal profiles, with shotgun metagenomics as a reference. In addition, we developed a custom text mining-based filtration approach to facilitate species-level annotations from amplicon sequencing data.

## Methods

### Sample collection and processing

We sampled 50 non-pregnant Caucasian women aged 25–45 years attending population-based cervical cancer screening as previously described [33]. The exclusion criteria were vaginal intercourse 48 h prior to sampling, pregnancy, previous hysterectomy, and the inability to tell the time of the last menstrual period. Detailed subject characteristics have been previously reported [33]. All samples were collected with a sterile flocked swab (FLOQSwabs, Copan spa, Italy) from the right fornix of the vagina during a speculum exam. DNA extraction was done using bead beating as described [33]. The extracted DNA was stored at − 20 °C.

### Sequencing of 16S rRNA gene and Internal transcribed spacer-1 (ITS1) amplicons

Data generated from an earlier study [33] targeting the V3–V4 region of the 16S rRNA gene (PRJEB25778) was used as a control dataset to determine any data loss or variation that might be brought on by parallel sequencing. This sequencing run was done using paired-end mode (PE250 kit) and Illumina HiSeq as detailed previously [33]. For the parallel sequencing approach, the 16S rRNA gene and ITS amplicons were generated separately from the previously extracted frozen DNA and pooled for barcoding.

For bacteria, we used the same 16S rRNA gene primers for V3–V4 region as before, specifically 341F 5′-CCTACGGGNGGCWGCAG-3′ and 785Rev 5′-GACTACHVGGGTATCTAATCC-3′ [33, 34]. These primers have been validated to provide high taxon coverage for vaginal bacteria [35]. The reaction comprised of 5 ng/µl template, 1X Phusion® Master Mix (ThermoFisher, catalogue number: F-531L), 0.375 µM V3–V4 locus-specific primers and DMSO. The PCR was run under the following settings: 98 °C for 60 s, 40 cycles of 98 °C for 10 s, 64 °C for 40 s, 72 °C for 40 s and finally 10 min at 72 °C, whereafter the samples were stored at 4 °C.

For fungi, a two-step PCR was applied due to the poor amplification when using amplicon-specific primers with overhang adapters. PCR-amplicons of the ITS1 region were generated using ITS1F and ITS2 primers (5′-GGTCATTTAGAGGAAGTAA-3′ and 5′-GCTGCGTTCTTCATCGATGC-3′ [36, 37]. The primers for the second PCR to add the adapters were adapted from [38]. Both ITS PCR reactions were conducted in a total reaction volume of 20 µl consisting of 10 µl of 1 × Phusion® Master Mix, 4.4 µl of the template, 0.5 µl of each primer, 0.6 µl of DMSO, with water added to reach the final volume. Template concentration at the first ITS PCR was 22 ng of DNA and at the second the amplicon derived from the previous PCR step was used. The cycling parameters for both ITS PCRs were 98 °C for 60 s, followed by 44 cycles of 98 °C for 10 s, 58 °C for 40 s, 72 °C for 40 s and finally 10 min at 72 °C, after which the samples were stored at 4 °C.

Both 16S rRNA gene and ITS1 PCR products were visualized using agarose gel electrophoresis and then purified with AMPure XP beads (Beckman Coulter, Copenhagen, Denmark). After purification, both PCR products were quantified using Quant-it PicoGreen dsDNA assay kit, 2000 assays (Invitrogen, cat.nro P7589). The index PCR was performed to add the barcoded primers [38] for sequencing, which was done using the following settings: 98 °C for 60 s, 30 cycles of 98 °C for 10 s, 64 °C for 30 s, 72 °C for 30 s and finally 10 min at 72 °C. The 16S rRNA gene amplicons were diluted to 40 nM and the two locus-specific PCR products were mixed 1:1 for volume to be used as a template. The PCR amplification was performed with 4 µl template, 10 µl 1 × Phusion® Master Mix, 0.5 µM each barcoded primer, 0.6 µl DMSO, with water added to reach the final volume of 20 µl.

**Fig. 1:**
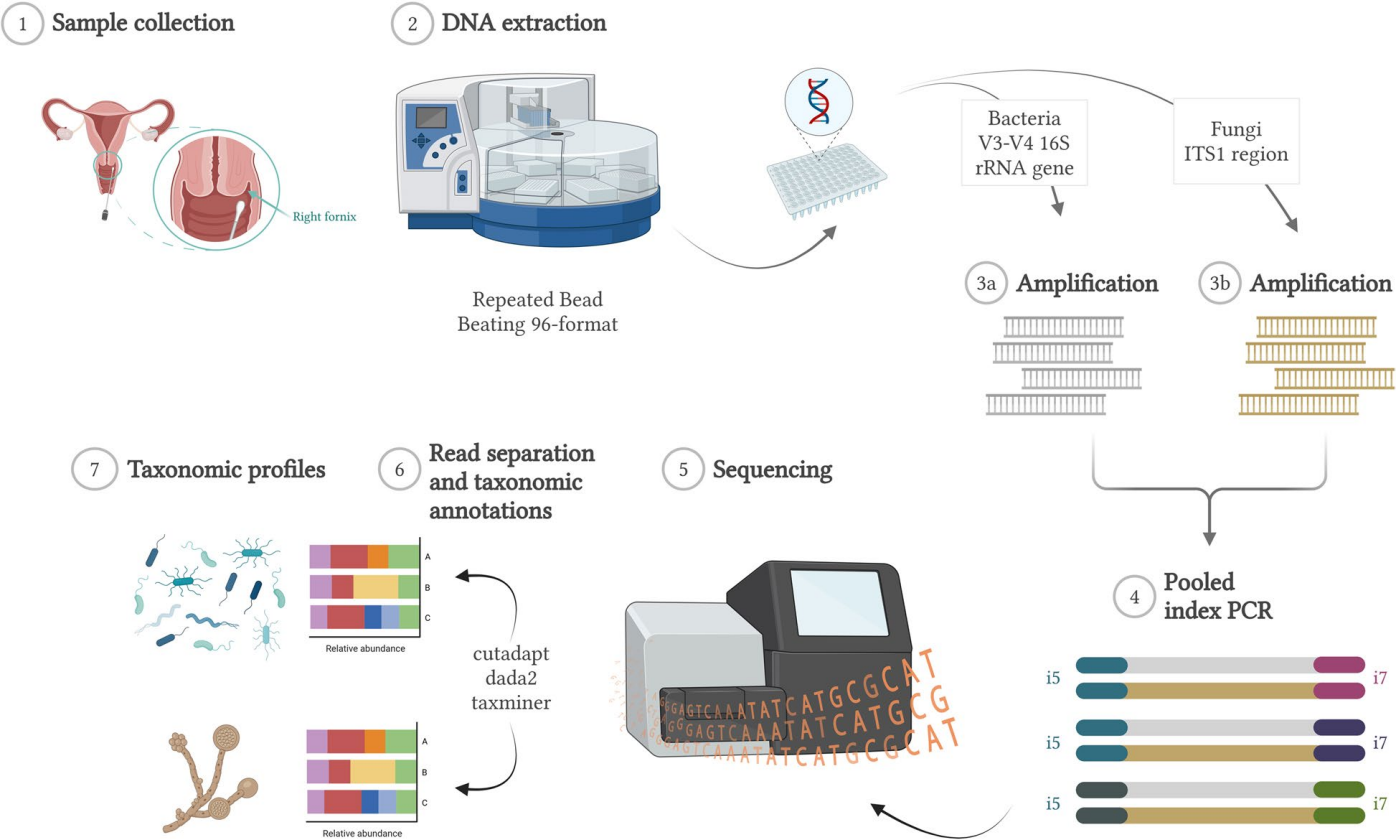
Illustration of the general workflow utilized within this study. Created with BioRender.com. Annotation workflow for obtaining the taxonomic profiles is presented in Fig. 2

The PCR products were quantified using Quant-it PicoGreen dsDNA assay kit (Invitrogen, cat.nro P7589). A 50 nM pool was prepared, and its clean-up was performed with AMPure XP beads (Beckman Coulter, Copenhagen, Denmark). It is important to ensure that the bead purification step efficiently removes primer dimers and/or ITS1 amplicons shorter than the read length as those have negative effects on the run performance. The pooled 16S rRNA gene V3–V4 and ITS1 amplicon mixture was sequenced at the Biomedicum Functional Genomics Unit (FuGU), Helsinki, Finland with an Illumina MiSeq instrument using paired end 2 × 300 bp reads and a MiSeq v3 reagent kit. The loading concentration was 7.5 pM with a 15% PhiX spike-in. The sequencing workflow is illustrated in Fig. 1.

### Pre-processing and splitting of 16S rRNA gene and ITS1 reads

First, we removed reads with ambiguous (Ns) bases and used cutadapt version 2.6 to trim the primers from raw reads and to split them into separate 16S rRNA gene and ITS1 amplicon datasets (fastq-files) with ‘discard_untrimmed’ option [39]. With this method, the 16S rRNA gene or ITS1 library could be extracted from the mixed library and analysed independently. We used the same pre-processing steps for both sequencing runs including the library split. After the library split, removal of primers, and ‘Filter and Trim,’ the two 16S rRNA gene libraries were pre-processed together with the DADA2 package version 1.12.1 in R version 3.6.1, following the DADA2 Pipeline Tutorial (1.12) [40, 41, 42]. The ITS1 library was pre-processed as in DADA2 ITS Pipeline Workflow (1.8) except the removal of primers and ambiguous bases was already done during the library split [43].

### Taxonomic assignment

**Fig. 2:**
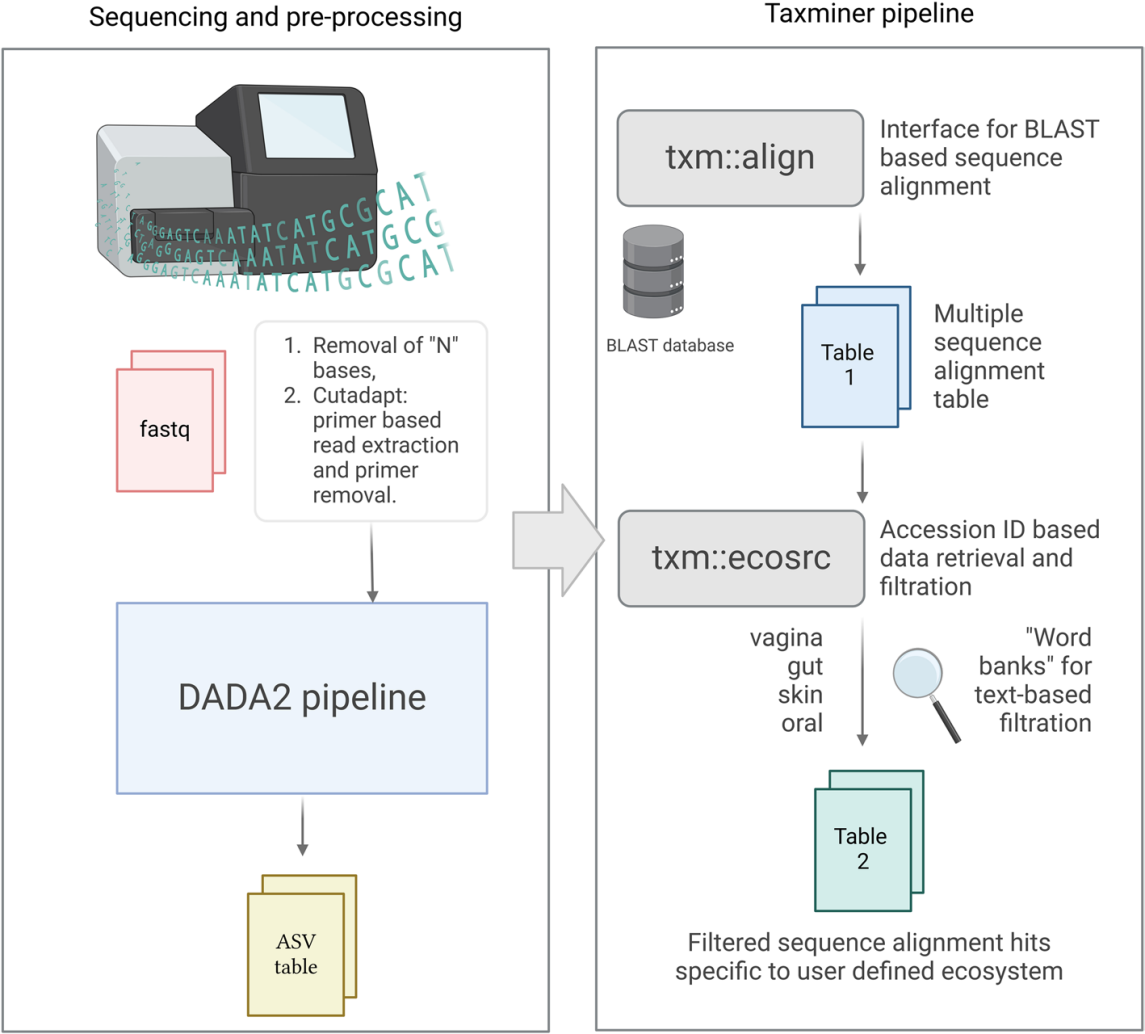
Workflow illustrating the main steps in the taxminer based taxonomic annotations. Created with BioRender.com

Of the tools available to us, we selected the BLAST alignment tool coupled with the NCBI nucleotide database to obtain the largest set of species-level assignments and implemented the ‘taxminer’ tool to automate the post-alignment selection between multiple sequence hits [44]. The tool supplies accession numbers for each hit to the rentrez R [45] package to communicate with the extensive network of NCBI databases and extract valuable background information for each annotation. The tool is comprised of two primary functions:

1. Txm_align. The main function for sequence alignment. It is primarily designed to work seamlessly with the dada2 pre-processing pipeline, using the resulting Amplicon Sequence Variant (ASV) table as the input and perform 5 main tasks. (1) The sequences are converted into FASTA format and written into a temporary folder. Optionally, if the input variables ‘batch’ and/or ‘chunk’ are specified, ‘seqkit’ [46] is used to split the FASTA file into smaller subsets for memory efficiency. (2) The sequences are aligned using BLAST (default: ‘megablast’) against the specified database, resulting in a tabular (outfmt 6 ‘qacc saccver staxids sscinames bitscore evalue qcovs pident’) output. GNU parallel [47] is used to align any specified batches/chunks and circumvent some memory requirements for larger queries and databases. (4) An optional ‘alt_annot’ step, uses the dada2 functions ‘assignTaxonomy’ (minBoot = 80) and ‘addSpecies’ (allowMultiple = T) to assign taxonomies from SILVA, RDP, and/or UNITE databases. These are further used to create a ‘score’ based on the taxonomic annotation consensus between the different approaches, which serve as a secondary post-alignment filter. (5) Taxonomic IDs are used to extract the lineage (super-kingdom to species) with ‘rentrez::entrez_fetch’ and combined into a final output table.
2. Txm_ecosrc. Primary function for extracting, processing, and compiling background information for each annotation hit. Accession and PubMed IDs are supplied to ‘rentrez::entrez_fetch’, obtaining information about the host, isolation source, publication title, and abstract for each hit. In the presence of alternative annotations (silva, rdp, or unite), scores are calculated, with higher weight applied to species-level consensus between tools/databases, coupled with known host and isolation sources, and published works. A collection of queries termed ‘Word banks’ are provided within the tool, which represents specific host organisms and human body sites that can be used as a filter to select the most likely alignment hits for each sequence. The primary workflow for taxonomic assignment is illustrated in Fig. 2. This tool can be flexibly used to stack different sets of word banks in succession to gain the maximum number of annotations between interrelated ecosystems, resulting in varying degrees of relevance and confidence. This trickle-down approach prevents oversaturated entries from masking actual results in a pool of multiple hits and applies a hierarchy between different ecosystems that may be relevant to the study. In our study, we stacked the vaginal, gut + skin, and clinical word banks. To ensure the veracity of the tool, the selected annotations were manually verified and confirmed.

### Statistical methods

The R package vegan (2.5–7) was used to calculate: (1) alpha diversity (Shannon), rarefaction, and (2) permutational multivariate analysis of variance (PERMANOVA) through the ‘adonis’ function to quantify the variance explained by the sequencing run type and other technical as well as clinical and other host variables on the bacterial and fungal composition within the samples [48]. Principal coordinate analysis was done using R packages phyloseq (1.34.0) and vegan, and the figures were plotted with ggplot2 (3.3.3) [49, 50]. All beta-diversity measures were based on Bray–Curtis dissimilarity. To classify the samples into community state types (CSTs) based on the microbial compositions, we used a tool called VALENCIA (VAginaL community state typE Nearest CentroId clAssifier) [30]. All bar plots are created using ggplot2 and heatmaps are created with pheatmap [51]. Correlation analysis between bacteria and fungi was done using Spearman correlation using R package Hmisc function rcorr [52].

### Shotgun metagenomic sequencing

To validate the performance of the combined 16S rRNA gene + ITS amplicon sequencing, shotgun metagenomic sequencing using Illumina technology was carried out on a subset of 21 samples that had sufficient DNA left (min 300 ng). The libraries were prepared using Illumina Nextera™ DNA Flex Library Prep Kit and sequenced on Illumina’s NovaSeq6000 S4 PE150 run. An additional metagenome dataset was created with ZymoBIOMICS Microbial Community Standard (Zymo, Irvine, CA, USA) and 16 vaginal samples representing different microbial profiles using MGI technology (kind support by Prof. Lars Engstrand and Maike Seifert; Karolinska Institutet, Sweden). Library preparation was carried out on 50 ng of genomic DNA with MGI´s FS library prep set (MGI, Tech Shenzhen, China) according to the manufacturer’s instructions. The quality of libraries was confirmed with the TapeStation D1000 kit (Agilent Technologies, Waldbronn, Germany) and the library quantity was assessed using QuantIT HighSensitivity dsDNA Assay on (Thermo Fisher Scientific, Waltham, MA, USA) using a Tecan Spark 10 M (Tecan Spark 10 M, Mannedorf, Switzerland). Circularized DNA of equimolarly pooled libraries was prepared using MGIEasy Circularization kit (MGI Tech). DNBseq HotMPS 150 bp paired-end sequencing was performed using the DNBSEQ T7 sequencing instrument (MGI Tech) according to the manufacturer’s instructions.

These samples were selected based on the detection of *Candida, Malassezia, Saccharomyces and Cladosporium* spp. in different proportions through ITS1 amplicon sequencing. Additionally, three control samples were added for which we failed to identify any fungi with ITS1 amplicon sequencing.

To evaluate the accuracy of the text mining-based annotation method on the 16S rRNA gene and ITS1 amplicon data, the metagenome data was analysed with several approaches using MetaPhlAn 3.0, Kraken 2 (with PlusPF database 17/05/2021 and Bracken v2.7), and VIRGO [17, 53, 54]. Finally, functional profiles were obtained using the HUMAnN 3.0 pipeline [55]. Briefly, reads per kilobase (RPK) values were converted to counts per million (CPM), using the renormalization utility script (‘humann_renorm_table’). Gene families were regrouped in level-4 enzyme commission (EC) categories using the regrouping utility script (‘humann_regroup_table’), and further combined with pathway abundance files using the unpacking script utility (‘humann_unpack_pathways’). Log-transformed CPM values were visualised as heatmaps with complete linkage clustering. The KEGGREST utility was used to extract further information associated with each EC number, i.e. enzyme name, reaction, and substrate [56].

## Results

### High concordance for bacterial data between the combined 16S rRNA gene + ITS1 run versus conventional 16S rRNA gene amplicon sequencing

The main aim of this study was to determine the feasibility and performance of a combined method of sequencing for bacterial 16S rRNA gene and fungal ITS1 region amplicons. Since the 50 vaginal samples were already analysed in a previous publication for the bacterial compositions, the existing 16S rRNA gene amplicon data and the results obtained therein serve as a point of reference [33]. Both the reference and combined sequencing runs were analysed separately to address their individual pre-processing requirements, followed by taxonomic annotations using the pipeline described in the Methods. A total of 48/50 samples that met the quality thresholds were kept for further analysis and comparisons. Upon primer-based separation, the combined run contained a mean of 38,321 (32%) and 82,504 (68%) reads for 16S rRNA gene and ITS1 region amplicon sequences. As expected, a majority of reads in the reference run were 16S rRNA gene amplicons (mean 53,098, 98%); however, a negligible amount of ITS1 amplicons was also detected (mean 780, 1%). Further details of both sequencing runs can be found in Fig. 1 and Supplementary file S1–S2.

**Fig. 3:**
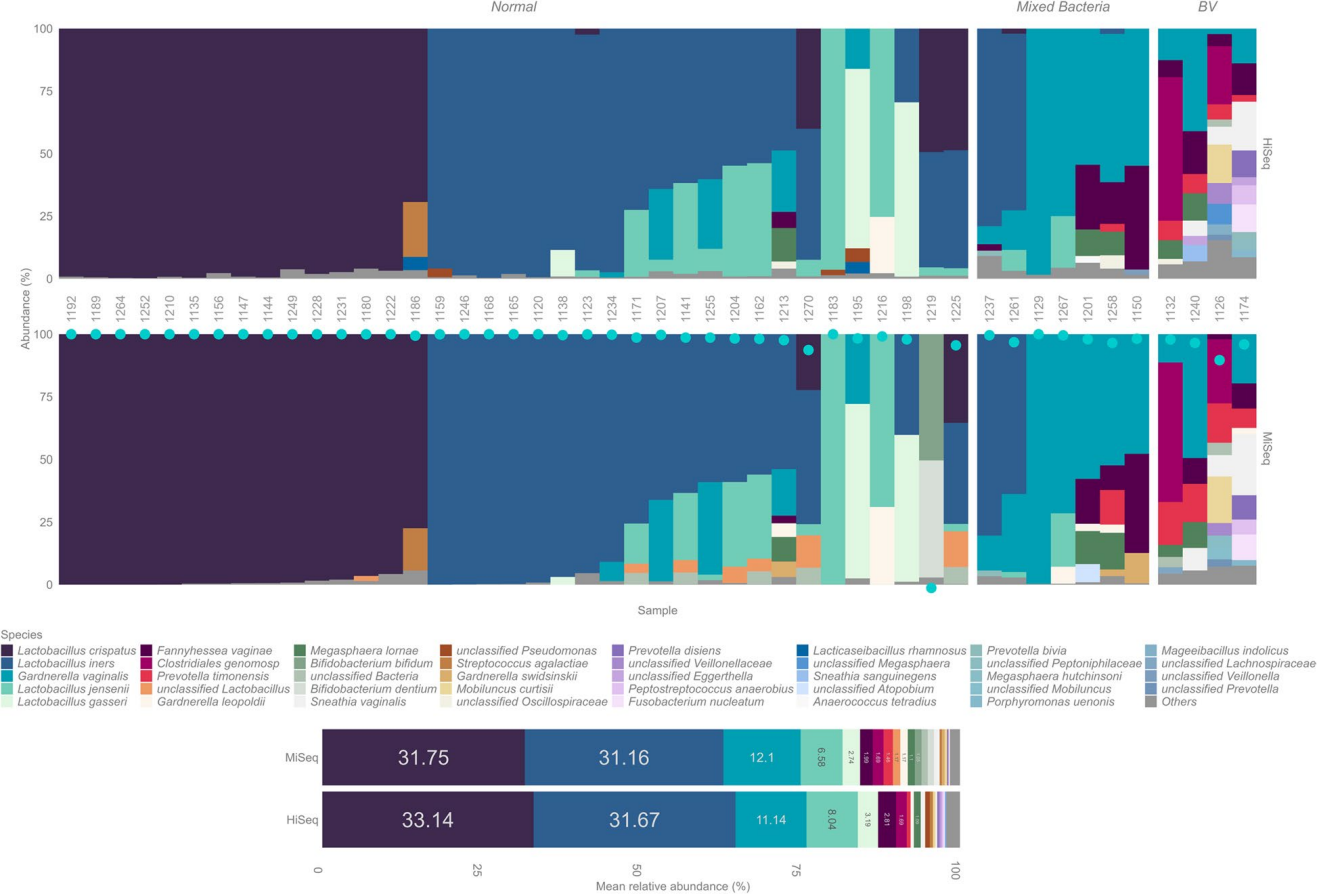
Bacterial relative abundances. Comparison of bacterial compositions derived from the combined 16S rRNA gene + ITS1 amplicon run (MiSeq, lower panel) versus a conventional 16S rRNA gene amplicon sequencing run (HiSeq, upper panel). Cyan dots represent the per sample Pearson’s correlations between the bacterial profiles derived from the two run types

In terms of bacterial profiles, the two runs were highly similar (Pearson’s correlation 0.97), a vast majority of the samples clustered together in Principal coordinate analysis (PCoA) plots (Fig. S1), and negligible variance was explained by the run type in PERMANOVA (*R*^2^ = 0.004, *p* = 0.8). As previously published [33], lactobacilli were the most prevalent bacteria (Fig. 3). *L. iners* was detected at > 5% abundance in 20/48 samples and *L. crispatus* in 17/48 samples in both the reference and combined run. For lower abundance lactobacilli, we detected *L. jensenii* (Reference 10/48, combined 7/48) and *L. gasseri* (3/48 and 2/48). One sample (1219) was a complete outlier in the reference run compared to the rest of the analyses (combined amplicon and WGS runs) and was not included in these numbers.

Three *Gardnerella* species were observed above 5% abundance, of which *Gardnerella vaginalis*, associated with bacterial vaginosis was the most prevalent (Reference: 14/48, Combined: 15/48). Other species observed were *G. leopoldii* (Reference: 1/48, Combined: 3/48) and *G. swidsinskii* (Reference: 0/48, Combined: 2/48). *Fannyhessea vaginae* (formerly known as *Atopbium vaginae*) was seen to co-occur with *G. vaginalis* (Reference: 7/48, Combined: 5/48)*.* We also observed *Clostridiales genomosp.* (CP049781.1)/*Candidatus Lachnocurva vaginae* in non-*Lactobacillus*-dominated samples that were microscopically assessed to be composed of BV-associated bacteria [18]. This bacterium was previously known as Bacterial vaginosis-associated bacterium 1 (BVAB1) and has recently been renamed as ‘*Candidatus Lachnocurva vaginae*’ [32]. When the ASVs for this bacterium were annotated against the Silva database, they were assigned as *Shuttleworthia*, which has previously been shown as a mis-annotation [31, 32]. Full bacterial composition summaries for both runs can be found in Fig. 3 and Supplementary file S2.

## Vaginal mycobiota based on ITS1 amplicon sequencing

**Fig. 4:**
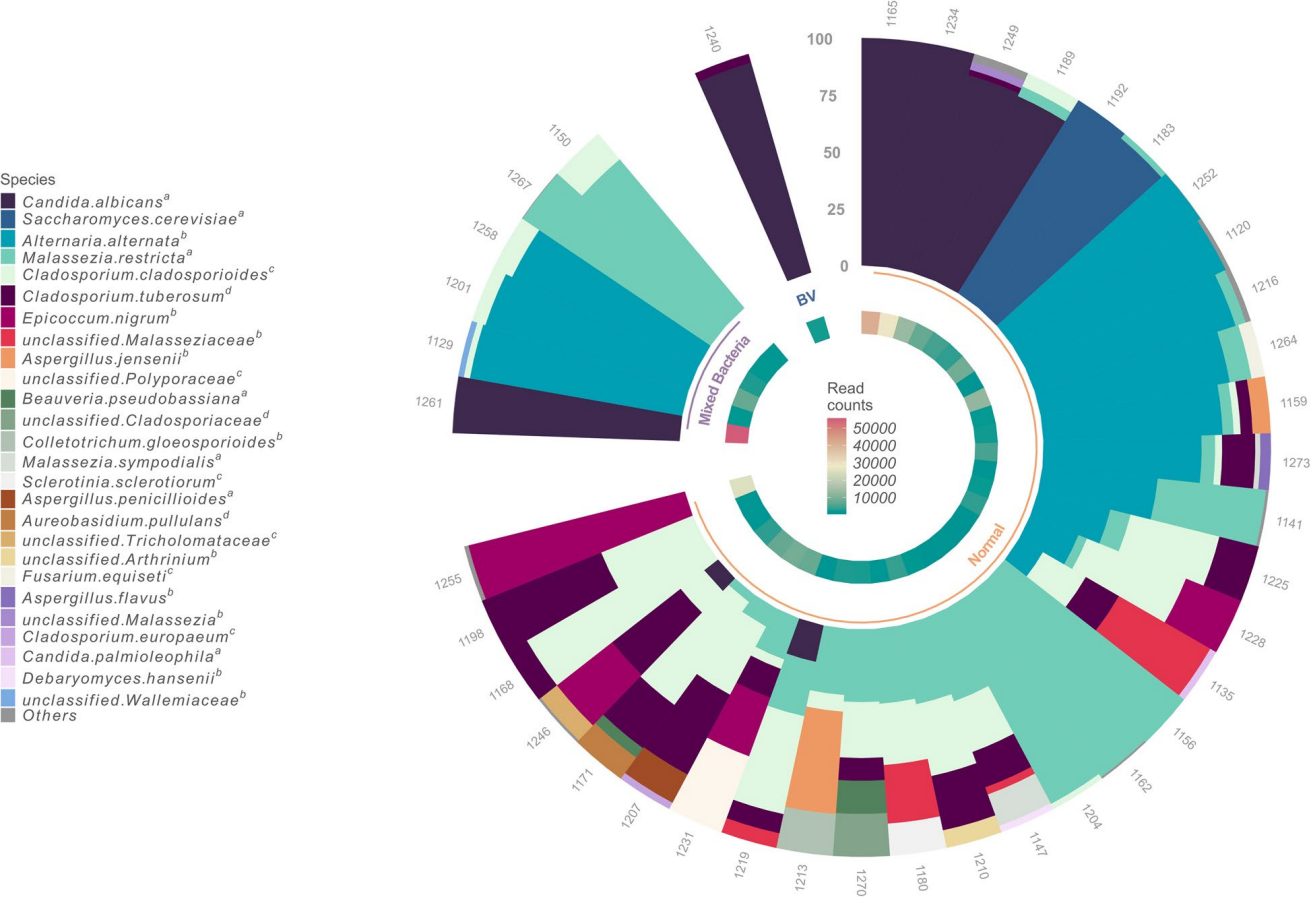
Fungal relative abundances. Fungal profiles from the combined 16S rRNA gene and ITS amplicon sequencing run. Taxa in the legend are labelled with the scoring groups of the taxminer tool, representing congruence with taxonomic annotations obtained from UNITE. ^a^ = species level overlap, ^b^ = genus level, ^c^ = family level, and ^d^ = lower than family level overlap

From the ITS1 reads of the combined run, 39/50 (78%) samples met the minimum read count threshold of 500 for processed and successfully annotated fungal reads (mean 5700 reads) and in those samples, 58 ASVs were successfully annotated (98% query coverage and percentage identity) (Fig. 4). The ASVs were further agglomerated to the species level, resulting in 29 unique fungal annotations. In the negative control sample, vast majority of the raw reads were shorter than 50 bases (peaks at 35 and 49 bases) and removed after library split and dada2 analyses so that there were only 10 ITS reads and 6 16S reads. The most commonly detected phylum was the Ascomycota (20/39 samples), with the most prevalent species being *Candida albicans* (8/39), *Alternaria alternata* (13/39), *Cladosporium* species (*C. tuberosum* 14/39, *C. cladosporiodes* 18/39), *Epicoccum nigrum* (4/39), and *Saccharomyces cerevisiae* (2/39) (Fig. 4). The Basidiomycota, represented by 9/29 species, in this dataset were primarily *Malassezia*, with the most prevalent being *M. restricta* (17/39), unclassified *Malasseziaceae* (3/39) and *M. sympodialis* (1/39).

**Fig. 5:**
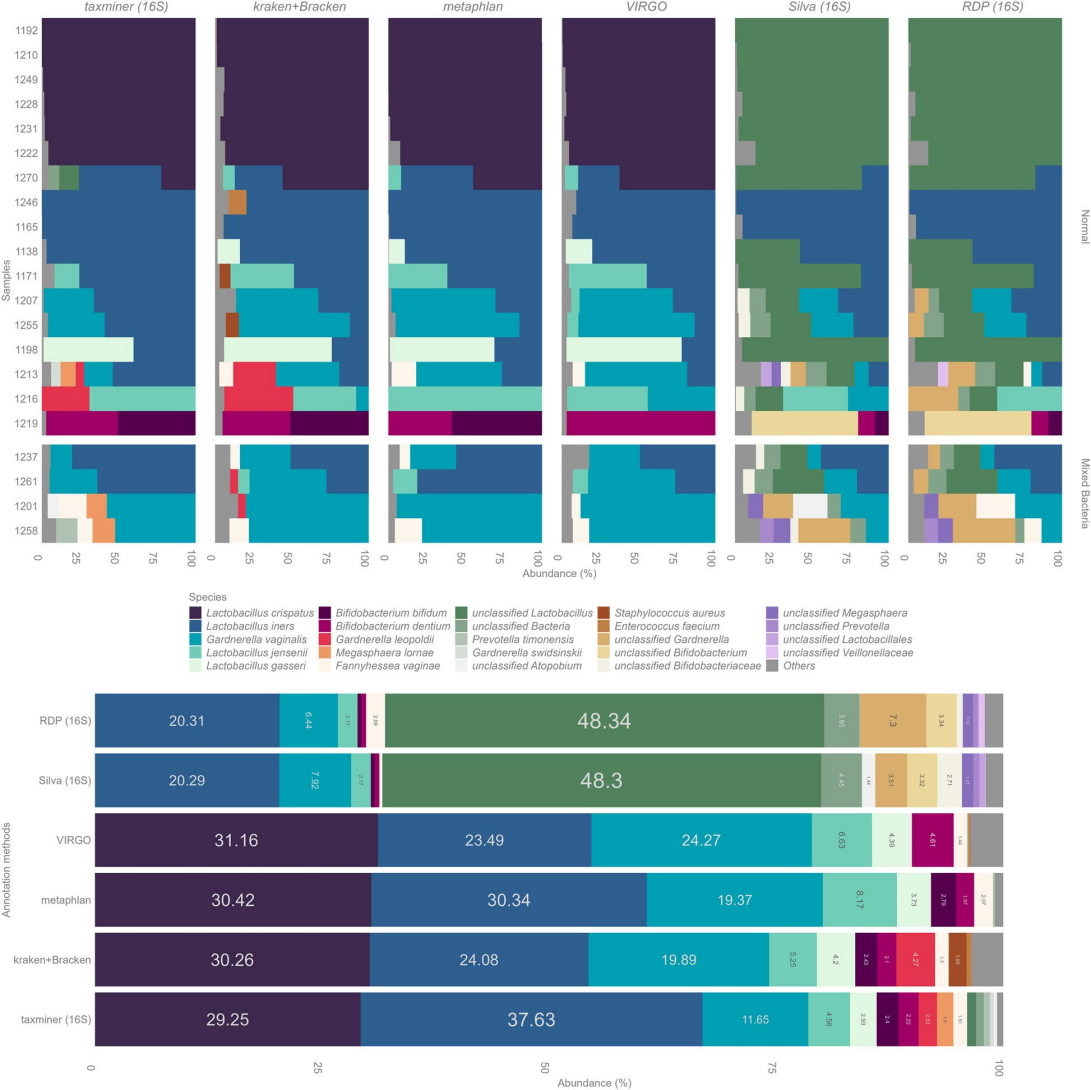
Comparison of the bacterial compositions derived from different runs, annotation tools and databases for a subset of 21 vaginal samples. Data from the combined 16S rRNA gene + ITS1 sequencing was annotated with the taxminer pipeline and dada2 ‘assignTaxonomy’ with the Silva and RDP databases. Shotgun metagenome data (Illumina) of the same samples was annotated with MetaPhlAn, Kraken + bracken (w/ PlusPF database) and VIRGO database as indicated on top of the panels

A total of 34 technical and host-related variables were tested using PERMANOVA analysis against the fungal profiles. Technical variables such as read counts and diversity as well as the number of sexual partners (lifetime) were the only ones that were statistically significant (Supplementary Fig. S2). Due to the low prevalence of even the most abundant fungal species a larger cohort is needed to study associations between background factors or clinical phenotypes and the mycobiota. To determine the co-occurrence patterns between vaginal bacteria and fungi, the abundance profiles of microbes that made up at least 1% of a sample and were present in 10% (5 samples) of the dataset were filtered and correlated (Fig. 5). We observed no significant correlations between the dominant lactobacilli—*L. crispatus* and *L. iners—*and fungi. However, *L. jensenii* and an *unclassified Lactobacillus* were positively correlated to *M. restricta*. Other correlations of note were the negative correlation between *G. vaginalis* and *C. tuberosum* and the positive correlation between *C. cladosporioides* and a group of unclassified bacteria.

## Metagenomics results in relation to the 16S rRNA gene and parallel 16S rRNA gene + ITS1 amplicon sequencing

To assess the performance and taxonomic coverage of the parallel 16S rRNA gene + ITS1 sequencing, we compared the taxonomic outputs to metagenomic shotgun sequencing. We also needed to benchmark the text-mining-based annotation pipeline against an independent method of species identification. The 21 libraries consisted of a total 557,102,162 read pairs 2 × 150 (per sample average 26,528,674; min 10,276,162; max 46,160,072). Overall, there was high concordance within the bacterial compositions across all sequencing runs and annotation methodologies (Fig. 5, Fig. S3 (correlation heatmap), PERMANOVA between annotation methods (taxminer, MetaPhlAn, kraken and Virgo) *R*^2^ = 0.026, *p* = 0.70). We found no falsely annotated species among abundant and named (cultured) species with our 16S rRNA gene annotation pipeline compared to clade-specific metagenomic species annotation (MetaPhlAn), the k-mer-based Kraken2 annotations, or the vaginal-specific VIRGO database. Some *Gardnerella* species, namely, *G. leopoldii* and *G. swidsinskii,* were only detected in the Kraken2 and amplicon results, primarily due to the up-to-date databases used for annotations. *Bifidobacterium bifidum* was not observed in VIRGO annotations while being dominant in one sample (1219) in all other data sets.

Fungi could not be reliably detected through metagenomics either with MetaPhlAn or Kraken2, which use clade-specific marker genes and k-mer compositions for taxonomic assignments, respectively. In kingdom-level analysis the yield for fungal reads was only 0.08–0.17% and the fungal community compositions could not be determined except *S. cerevisiae* was detected in a single sample (ID: 1192), and *C. albicans* in sample 1165 were exclusively dominated by these taxa in the ITS1 amplicon data. We used the PlusPF database for Kraken-based taxonomic annotations as it is one of the most extensive databases available, containing both bacteria and fungi. We observed that the eukaryotic reads that were left on the metagenomic data set after removal of human reads were predominantly annotated as ‘*Toxoplasma gondii*,’ a parasitic protozoan, which is a highly unlikely true hit given the characteristics of our study cohort. To investigate further, we extracted the reads that were annotated as *T. gondii* and aligned them against a custom, fungi-only Kraken database. While many annotated to *Malassezia* and *Candida* species, a considerable proportion of these reads remained ambiguous and made it difficult to define the fungal profiles. We further attempted to identify fungi in the same sample set with deeper metagenome data generated with the MGI technology, consisting of an average of 75 million reads per sample, of which an average of 0.01% of reads could be annotated as fungal reads when using the PlusPF database, and up to 0.20% of reads when using a custom fungal only database. Dominant and well-known fungal species, *C. albicans* and *S. cerevisiae,* could be confirmed in three metagenome samples using Kraken2 with the PlusPF and custom fungal-only databases. For the remaining 13 metagenome samples, the majority of fungal reads were annotated as *Aspergillus flavus*, which is an unlikely true finding for vaginal samples [57]*.* Additionally, we attempted to acquire annotations with FunOMIC, a fungal-specific metagenome analysis tool [58], and got coherent results with Kraken2 and ITS1 results for the three samples dominated by *C. albicans* or *S. cerevisiae.* However, fungal reads from the mock community containing 2% of two fungi, *S. cerevisiae* and *Cryptococcus neoformans* were almost entirely assigned to *S. cerevisiae* (Supplementary Fig. S4)*,* and for the rest of the vaginal samples, the results were random. Kraken2 performed well with the fungal part of the mock community but was biased towards bacterial compositions (Supplementary file S3). In summary, while the number of reads assigned to fungi was significantly higher, we could not obtain more reliable and reproducible fungal profiles from the deeper metagenome sequencing. We did not use the VIRGO database for fungal annotations because it is restricted to *Candida* spp.

## Functional attributes of the vaginal microbiota

After the removal of the human reads, functional profiling with the HUMAnN pipeline identified 104 metabolic pathways and 381 enzymes, revealing the metabolic potential of the bacteria present within these samples (*n* = 21). Hierarchal clustering of the functional profiles grouped samples with similar bacterial compositions and resultant CSTs (Supplementary Fig. S5), suggesting major differences in the metabolic potential of the prevailing bacterial community types in the vagina. For a full list of individual pathways see Supplementary Figs. S6–S7. Further results are shown and discussed in Supplementary document S1.

## Discussion

### Performance and output of the combined 16S rRNA gene and ITS amplicon sequencing

To our knowledge, at the time of conception, realisation, and initial submission of the manuscript (available online as a pre-print since 2021 here: https://doi.org/10.21203/rs.3.rs-321778/v1), our combined sequencing method was a novel approach to simultaneously obtain bacterial and fungal taxonomic profiles for clinical samples from a single sequencing run. However, during the review and revision process, another article was published with a similar approach for buccal samples collected from four intubated ICU patients [26]. Song et al. used different target regions for bacterial and fungal DNA and performed the annotations without splitting the data into fungal and bacterial reads. Previously, Kittelman et al. reported a pyrosequencing approach that combined analysis of phylogenetic marker genes for bacteria, archaea, and eukaryotic micro-organisms in ruminal samples, and Coller et al. combined 16S rRNA gene and fungal ITS1 amplicons to an Illumina MiSeq run to study soil samples [59, 60]. In addition, many studies have generated 16S rRNA gene and fungal ITS amplicons in the same samples, but the analyses were done separately [13, 14, 19, 20, 22, 23, 24, 25, 61, 62]. Kits for single library constructions for bacteria and fungi are also commercially available (at least from Swift, Qiagen and Perkin Elmer), but the costs per sample are substantially higher and hence our method provides a cost-effective option for laboratories that process high sample volumes and possess the basic expertise for library preparation for multiplex sequencing [59, 60]. Our protocol where the pooling of bacterial and fungal amplicons takes place already before indexing (after the locus-specific PCRs), saves time and costs as all downstream steps occur in a single well. The use of the same barcodes for bacterial and fungal amplicons further simplifies the design and improves the efficiency especially when there are a limited number of barcodes available. Overall, the method provides combined profiling for both bacteria and fungi with little added cost compared to ordinary 16S rRNA gene sequencing. The 16S rRNA gene and ITS reads can be separated easily with publicly available bioinformatic tools like cutadapt that were used here and were the only additional step in pre-processing. Comparisons to conventional 16S rRNA gene amplicon and metagenomic sequencing demonstrated that the newly developed method produced highly comparable results for bacterial compositions and there was a high degree of reproducibility between the results despite some methodological differences (HiSeq vs MiSeq, PE250 vs. PE300 chemistry) as possible source of variation. It should be noted that for successful simultaneous analysis of bacterial and fungal communities from the same sample, also efficient cell disruption is required to retrieve representative DNA for further analysis. We used a bead-beating method for DNA extraction that has been previously shown to perform well in disrupting the rigid cell walls of fungi [63]. It should be noted that when setting up the system, it is advisable to test whether the chosen sample type and/or PCR mastermix will allow for a lower number of PCR cycles without compromising the PCR yields. For sample types that have fungal species with ITS1 regions substantially longer than the sequencing read length it is advisable to use the forward reads only or consider long-read sequencing.

#### Text-mining-based annotation and prioritization of hits

At present, most amplicon sequencing workflows employ sequence homology as the primary criterion for taxonomic classification that especially with partial 16S rRNA gene sequences may fail in reliable species-level identification. Here, we obtained taxonomic annotations from an exhaustive nucleotide database and combined them with deposited background information to automate the post-alignment curation of sequence alignment hits. Through this approach, species-level annotations could be extracted from 16S rRNA gene amplicon sequencing that were comparable to bacterial profiles obtained from WGS sequencing. High concordance with the vaginal microbiota-specific VIRGO database further confirmed the accuracy of the bacterial profiles from a larger and unrestrained database.

By utilizing word banks for inclusion, taxminer automatically selects biologically plausible hits and weeds out mis-annotations, which are easily left unnoticed unless completely irrational [64]. While the well-described and relatively homogenous vaginal microbiota was profiled to species level with high confidence, more complex ecosystems such as the gut microbiota might require further curation. We acknowledge that this approach is tethered to the inherent limitations of the 16S rRNA gene amplicon sequencing as well as the quality and availability of information on public databases.

By utilizing word banks for inclusion, taxminer automatically selects biologically plausible hits and weeds out mis-annotations, which are easily left unnoticed unless completely irrational [64]. While the well-described and relatively homogenous vaginal microbiota was profiled to species level with high confidence, more complex ecosystems such as the gut microbiota might require further curation. We acknowledge that this approach is tethered to the inherent limitations of the 16S rRNA gene amplicon sequencing as well as the quality and availability of information on public databases.

#### Microbial profiles

Shotgun metagenomics (WGS) and conventional 16S rRNA gene amplicon sequencing validated the bacterial profiles obtained from our parallel sequencing approach. Since the samples were from healthy subjects, they were dominated by lactobacilli*,* while 11 samples with mixed/BV-like microscopy results [33] were populated with *G. vaginalis*, *F. vaginae*, *Veillonella spp.*, *uncultured bacteria*, and *C. genomosp*. *(BVAB1).* Since we report relative abundances, the discrepancy observed in the proportion of *G. vaginalis* observed in some samples between the methods could be attributed to the absence of uncultured bacteria from the metagenome results, which in the 16S rRNA data made up to 10% of the total abundance of the samples in question. The inability of the metagenome pipeline to detect low prevalence and/or uncultured bacteria is largely due to incomplete reference databases.

While we obtained high concordance between the bacterial profiles obtained with our combined amplicon sequencing vs. WGS approach, fungi beyond the well-known *C. albicans* and *S. cerevisiae* could only be detected with the former. This is due to the low abundance of fungal reads within the metagenomes, their poor coverage in the reference databases used for the WGS approach as well as apparent contaminants or mis-annotations of the reference sequences [65]. A further challenge in metagenome-based taxonomic annotations is large, non-dedicated databases, such as the pre-compiled PlusPF database from Kraken2, where fungal reads may be sequestered by other (micro) eukaryotes, which highlights the need for alternative methods to reliably identify fungal communities in host-associated ecosystems. The yield for fungal reads within the vaginal metagenomes was 0.08 to − 0.17% in our study and 0.17 ± 0.04% in the VIRGO study [17]. Eukaryotic genomes are also significantly larger and much more complex compared to prokaryotes, which further hinders metagenome analysis, especially with a restricted set of reference databases [66]. Even with amplicon data that provide a more targeted approach for fungal profiling, the analysis of fungi is much more challenging than that of bacteria, e.g. due to the natural length polymorphism of ITS1 region that may complicate both the actual sequencing and subsequent bioinformatic analyses. Apart from *S. cerevisiae* being detected in two samples and *C. albicans* in one sample with deep sequencing, there were no consistent and/or biologically plausible positive hits in the WGS data while 29 unique fungal species were detected across the 50 samples by ITS1 amplicon sequencing. When considering the possibility of contamination or other artefacts of the ITS1 amplicon data, it should be noted that we used the same DNA extracts for both metagenomic and amplicon sequencing, and our negative, non-template PCR control yielded only 10 ITS1 reads after preprocessing compared to a mean of 5700 reads for vaginal samples. Moreover, the ITS1 primers have been used for two decades and represent the gold standard for the analysis of fungi. Their performance has been previously validated with mock communities [67, 68]. Moreover, 3/29 fungal species (*C. cladosporioides, A. alternata, E. nigrum*) that we detected with ITS1 sequencing, have previously been cultured from the cervicovaginal samples of healthy giant pandas [69]. Representativeness of all the prevalent (≥ 10%) fungal genera detected in this study with the ITS1 amplicon data, i.e. *Candida, Cladosporium, Malassezia, Alternaria and Epicoccum* were also detected in the single previous study so far using ITS1 amplicons to investigate the mycobiota in human vaginal samples [13], as well as in the perianal and genital area of infants using ITS2 amplicons [70]. Other fungal studies on human vaginal samples so far have used ITS2 amplicons [14, 15, 16] or ITS (from ITS1 to ITS2) and each reported various non-*Candida* species despite various cell lysis and bioinformatic protocols. Together these results strongly support the view that the human reproductive tract mycobiota consists of various fungi, not just *C. albicans.* However, the fungal NGS results should be validated with independent methods.

In metagenome analysis, there were no clear benefits of deeper sequencing since we observed very similar fungal profiles, which confirmed our previous findings. Our results and conclusions on the higher accuracy and much better cost-effectiveness of fungal profiles obtained with ITS1 amplicon data compared to the WGS data are in line with previous papers on 1772 stool samples [71] as well as with a comprehensive study on bioaerosol fungi analysed through ITS1, ITS2, metagenome and culturing approaches [65]. Together these results show that even in this era of relatively low-cost shotgun metagenomic sequencing, amplicon sequencing still has the edge when studying complex low-abundance communities. On the other hand, large public WGS datasets can be used for the mining of bacteria of interest including pathogens to produce prevalence estimates [72], but for clinical studies, and especially pathogen-focused ones, separate validated and inexpensive testing protocols can be recommended instead or in addition to WGS detection.

With the lack of studies focusing on fungi, deposited fungal sequences with valid human isolation sources are also scarce. In our samples only *C. Albicans*, a well-characterized pathogenic yeast responsible for vulvovaginal candidiasis [5], was identified from vaginal samples. The remaining annotations were traced back to either the gut (*Aspergillus spp.*), skin (*Malassezia spp.* And *Cladosporium spp.*), or other clinical isolates (*S. Cerevisiae*, *Schizophyllum spp.*). Of these annotations, *Cladosporium spp.* was questionable due to the uncertainty surrounding their isolation sources. On one hand, the metadata for *Cladosporium spp.* annotation hits indicate that they were isolated from clinical human samples and certain species have been associated with human and animal infections [73]. On the other hand, they are also widely recognized as household fungi and are shown to be a consistent environmental contaminant in fungal studies [13, 74].due to the scarcity of information on the vaginal fungal communities and their potential associations to clinical and other host factors, we conducted the same permanova analysis for fungal profiles as previously done for the bacterial microbiota of the same samples [33]. We found no host-related variables to be strongly associated with the fungal abundance profiles while probiotic use and education were the strongest factors explaining bacterial variation [33]. Because of the small sample size of a healthy cohort, further studies with larger clinical and/or phenotypic variation are needed to study the potential interactions between the two kingdoms in the vaginal microbiota. To understand their ecology, information on ribosomal copy number variation and/or absolute abundances should be incorporated to estimate the abundance of each taxon more reliably.

## Conclusion

The main outcomes of this work are (1) development and validation of a novel cost-effective method for simultaneous amplicon sequencing for bacteria and fungi; (2) development of a novel annotation approach for microbial amplicon sequencing data where the conventional homology-based search is combined with text mining for knowledge-based selection of ecosystem-specific hits; (3) novel insights into the human vaginal ecosystem through the description of the mycobiota.

Our sequencing method should also be directly applicable to environmental sciences, agriculture, biotechnology, food technology, and any other field of science where studying bacterial and fungal populations is of particular interest. Due to the customizable nature of the text mining-based filtration approach, it can be adapted to a multitude of ecosystems under investigation.

## Supplementary Information

Supplementary Material 1.

Supplementary Material 2.

Supplementary Material 3.

Supplementary Material 4. Supplementary Figure S1: Principal coordinate analysis (PCoA) of the species level bacterial relative abundances.

Supplementary Material 5. Supplementary Figure S2: PERMANOVA results depicting the significance of background variables against the fungal profiles.

Supplementary Material 6. Supplementary Figure S3: Pearsons correlations between the species level bacterial relative abundances obtained from the different taxonomic annotations tools and databases.

Supplementary Material 7. Supplementary Figure S4: Fungal profiles obtained from the FunOMICs annotation tool.

Supplementary Material 8. Supplementary Figure S5: Community state types (CSTs) assigned to the bacterial profiles using the Valencia CST assignment tool.

Supplementary Material 9. Supplementary Figure S6: Heatmap illustrating the pathways identified in each sample through the Humann pipeline.

Supplementary Material 10. Supplementary Figure S7: Heatmap illustrating the pathways identified against taxa through the Humann pipeline.

Supplementary Material 11. Supplementary document 1: Results and discussion.

## Data Availability

The sequences have been deposited at the European Nucleotide Archive. ENA accession numbers: PRJEB25778 and PRJEB43447. The latest version of the R package ‘taxminer’ is available on GitHub (https://github.com/SchahzadSaqib/taxminer). The library split script has been added as a utility script to the same GitHub page.
