# Supplementary material for: Metagenome-validated combined amplicon sequencing and text mining-based annotations for simultaneous profiling of bacteria and fungi: vaginal microbiota and mycobiota in healthy women
Source: Microbiome. 2024 Dec 28;12:273. doi: 10.1186/s40168-024-01993-9 (PMC11681650; doi:10.1186/s40168-024-01993-9)
Supplement: Supplementary file 2 — Supplementary Material 1. [file 40168_2024_1993_MOESM1_ESM.zip › MiSeq27_ITS_readtrack_annot.html]

reactable


# MiSeq27 ITS readtrack annot
