# Supplementary material for: Metagenome-validated combined amplicon sequencing and text mining-based annotations for simultaneous profiling of bacteria and fungi: vaginal microbiota and mycobiota in healthy women
Source: Microbiome. 2024 Dec 28;12:273. doi: 10.1186/s40168-024-01993-9 (PMC11681650; doi:10.1186/s40168-024-01993-9)
Supplement: Supplementary file 3 — Supplementary Material 2. [file 40168_2024_1993_MOESM2_ESM.zip › Sequencing_summ.html]

|  |  |  |  |  |
| --- | --- | --- | --- | --- |
| Sequencing summary | | | | |
| Measure | Standalone 16S rRNA gene sequencing | | Combined 16S rRNA gene + ITS sequencing | |
|  | HiSeq (bac) | \*\*HiSeq (fungi) | MiSeq (bac) | MiSeq (fungi) |
| Raw input read counts | 54,292 (4,604 - 209,300) | 54,292 (4,604 - 209,300) | 120,472 (66,686 - 166,697) | 120,472 (66,686 - 166,697) |
| \*Filtered & separated read counts | 53,098 (4,448 - 205,205) | 780 (89 - 2,634) | 38,321 (9,778 - 86,404) | 82,504 (35,342 - 130,199) |
| % average reads (range) | 98 (96 - 98) | 1 (1 - 2) | 32 (9 - 61) | 68 (40 - 91) |
| dada2 output read counts | 45,793 (3,812 - 170,976) | NA | 27,809 (6,246 - 55,428) | 54,931 (5,211 - 106,289) |
| Succesfully aligned reads (BLAST) | 45,786 (3,812 - 170,839) | NA | 25,157 (4,470 - 54,899) | 8,083 (144 - 55,944) |
| Filtered & verified reads (taxminer) | 45,768 (3,812 - 170,832) | NA | 25,044 (4,370 - 54,752) | 5,834 (0 - 55,623) |
|  |  |  |  |  |
| --- | --- | --- | --- | --- |
| \* The reads containing ambiguous bases (Ns) were removed before the library split. | | | | |
| \*\* No PCR-amplification for ITS; likely reflecting cross-reactivity of 16S rRNA gene primers with 18S rRNA gene region upstream of ITS1. | | | | |
