# Supplementary material for: Metagenome-validated combined amplicon sequencing and text mining-based annotations for simultaneous profiling of bacteria and fungi: vaginal microbiota and mycobiota in healthy women
Source: Microbiome. 2024 Dec 28;12:273. doi: 10.1186/s40168-024-01993-9 (PMC11681650; doi:10.1186/s40168-024-01993-9)
Supplement: Supplementary file 4 — Supplementary Material 3. [file 40168_2024_1993_MOESM3_ESM.zip › KI_Kraken2_inventory.html]

KI metagenome reports


# KI Metagenome reports

#### Pavian R package v1.2.0

#### Wed Jul 5 16:18:11 2023

MGI DNBSEQ-T7 generated metagenomic data (kind support by Prof. Lars Engstrand and Maike Seifert; Karolinska Institutet, Sweden) for 16 samples was annotated with Kraken 2.0.8 at CSC – IT Center for Science, Finland. In this report we used four different databases:  
**A)** PlusPF database w/ raw data  
**B)** PlusPF database w/ decontaminted data  
**C)** Human associated fungal sequences extracted from the NCBI nucleotide database w/ raw data  
**D)** All taxa identified in the ITS amplicon sequencing w/ decontamintaed data

# Sample set summary

- Classification summary
- Raw read numbers
- Sample information

# Classification results

- Bacteria
- Viruses
- Eukaryotes
- Eukaryotes/Fungi
- Eukaryotes/Protists

Showing 100 of 7404 species.

Showing 100 of 155 species.

Showing 100 of 441 species.

Showing 100 of 399 species.

# Sankey visualization

## neg\_A

## neg\_B

## neg\_C

## neg\_D

## S1144\_A

## S1144\_B

## S1144\_C

## S1144\_D

## S1156\_A

## S1156\_B

## S1156\_C

## S1156\_D

## S1165\_A

## S1165\_B

## S1165\_C

## S1165\_D

## S1183\_A

## S1183\_B

## S1183\_C

## S1183\_D

## S1186\_A

## S1186\_B

## S1186\_C

## S1186\_D

## S1189\_A

## S1189\_B

## S1189\_C

## S1189\_D

## S1192\_A

## S1192\_B

## S1192\_C

## S1192\_D

## S1198\_A

## S1198\_B

## S1198\_C

## S1198\_D

## S1201\_A

## S1201\_B

## S1201\_C

## S1201\_D

## S1219\_A

## S1219\_B

## S1219\_C

## S1219\_D

## S1222\_A

## S1222\_B

## S1222\_C

## S1222\_D

## S1228\_A

## S1228\_B

## S1228\_C

## S1228\_D

## S1231\_A

## S1231\_B

## S1231\_C

## S1231\_D

## S1237\_A

## S1237\_B

## S1237\_C

## S1237\_D

## S1246\_A

## S1246\_B

## S1246\_C

## S1246\_D

## S1273\_A

## S1273\_B

## S1273\_C

## S1273\_D

## zymomock\_A

## zymomock\_B

## zymomock\_C

## zymomock\_D

# About

This file was generated with the Pavian R package version 1.2.0 on Wed Jul 5 16:18:17 2023. Please cite Pavian if you use it in your research.
