# Supplementary material for: Metagenome-validated combined amplicon sequencing and text mining-based annotations for simultaneous profiling of bacteria and fungi: vaginal microbiota and mycobiota in healthy women
Source: Microbiome. 2024 Dec 28;12:273. doi: 10.1186/s40168-024-01993-9 (PMC11681650; doi:10.1186/s40168-024-01993-9)
Supplement: Supplementary file 4 — Supplementary Material 3. [file 40168_2024_1993_MOESM3_ESM.zip › Kraken2_inventory.html]

Kraken2 metagenome reports


# Kraken2 metagenome reports

#### Pavian R package v1.2.0

#### Wed Jul 5 15:19:27 2023

Knead filtered metagenomic data for 21 samples was annotated with Kraken 2.0.8 at CSC – IT Center for Science, Finland. In this report we used four different databases:  
**A)** Microb (NCBI RefSeq bacterial, archea, viral, fungi and protozoa)  
**B)** PlusPF database  
**C)** NCBI fungi (all NCBI fungal sequences)  
**D)** Selected NCBI fungi (all NCBI sequences for fungal species found with ITS)  
For sample S1231 Kraken2 was unable to classify any reads with database D.

# Sample set summary

- Classification summary
- Raw read numbers
- Sample information

# Classification results

- Bacteria
- Viruses
- Eukaryotes
- Eukaryotes/Fungi
- Eukaryotes/Protists

Showing 100 of 5016 species.

Showing 100 of 290 species.

Showing 100 of 253 species.

# Sankey visualization

## S1138\_A

## S1138\_B

## S1138\_C

## S1138\_D

## S1165\_A

## S1165\_B

## S1165\_C

## S1165\_D

## S1171\_A

## S1171\_B

## S1171\_C

## S1171\_D

## S1192\_A

## S1192\_B

## S1192\_C

## S1192\_D

## S1198\_A

## S1198\_B

## S1198\_C

## S1198\_D

## S1201\_A

## S1201\_B

## S1201\_C

## S1201\_D

## S1207\_A

## S1207\_B

## S1207\_C

## S1207\_D

## S1210\_A

## S1210\_B

## S1210\_C

## S1210\_D

## S1213\_A

## S1213\_B

## S1213\_C

## S1213\_D

## S1216\_A

## S1216\_B

## S1216\_C

## S1216\_D

## S1219\_A

## S1219\_B

## S1219\_C

## S1219\_D

## S1222\_A

## S1222\_B

## S1222\_C

## S1222\_D

## S1228\_A

## S1228\_B

## S1228\_C

## S1228\_D

## S1231\_A

## S1231\_B

## S1231\_C

## S1237\_A

## S1237\_B

## S1237\_C

## S1237\_D

## S1246\_A

## S1246\_B

## S1246\_C

## S1246\_D

## S1249\_A

## S1249\_B

## S1249\_C

## S1249\_D

## S1255\_A

## S1255\_B

## S1255\_C

## S1255\_D

## S1258\_A

## S1258\_B

## S1258\_C

## S1258\_D

## S1261\_A

## S1261\_B

## S1261\_C

## S1261\_D

## S1270\_A

## S1270\_B

## S1270\_C

## S1270\_D

# About

This file was generated with the Pavian R package version 1.2.0 on Wed Jul 5 15:19:36 2023. Please cite Pavian if you use it in your research.
