# Supplementary figures and images for: Metagenome-validated combined amplicon sequencing and text mining-based annotations for simultaneous profiling of bacteria and fungi: vaginal microbiota and mycobiota in healthy women
Source: Microbiome. 2024 Dec 28;12:273. doi: 10.1186/s40168-024-01993-9 (PMC11681650; doi:10.1186/s40168-024-01993-9)

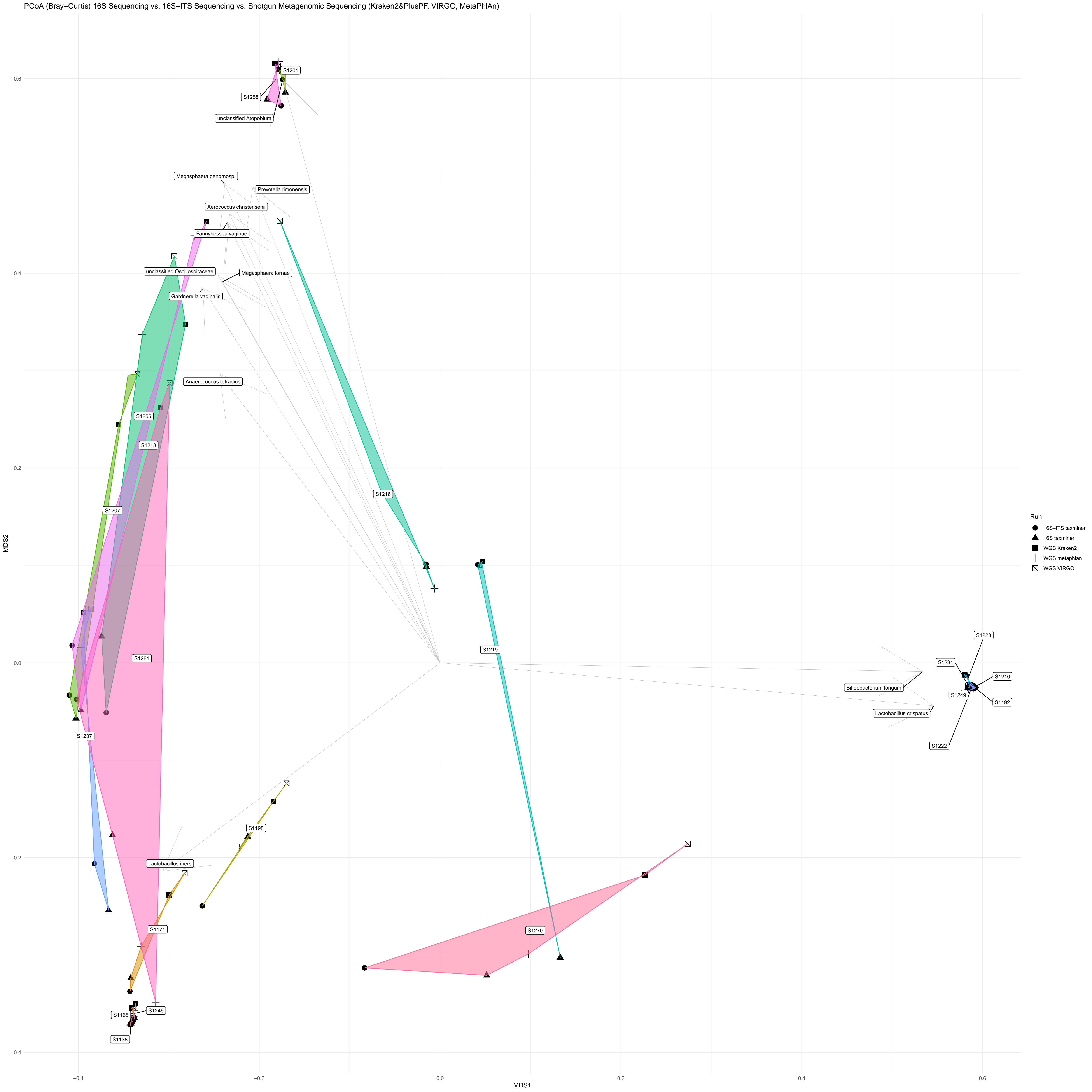

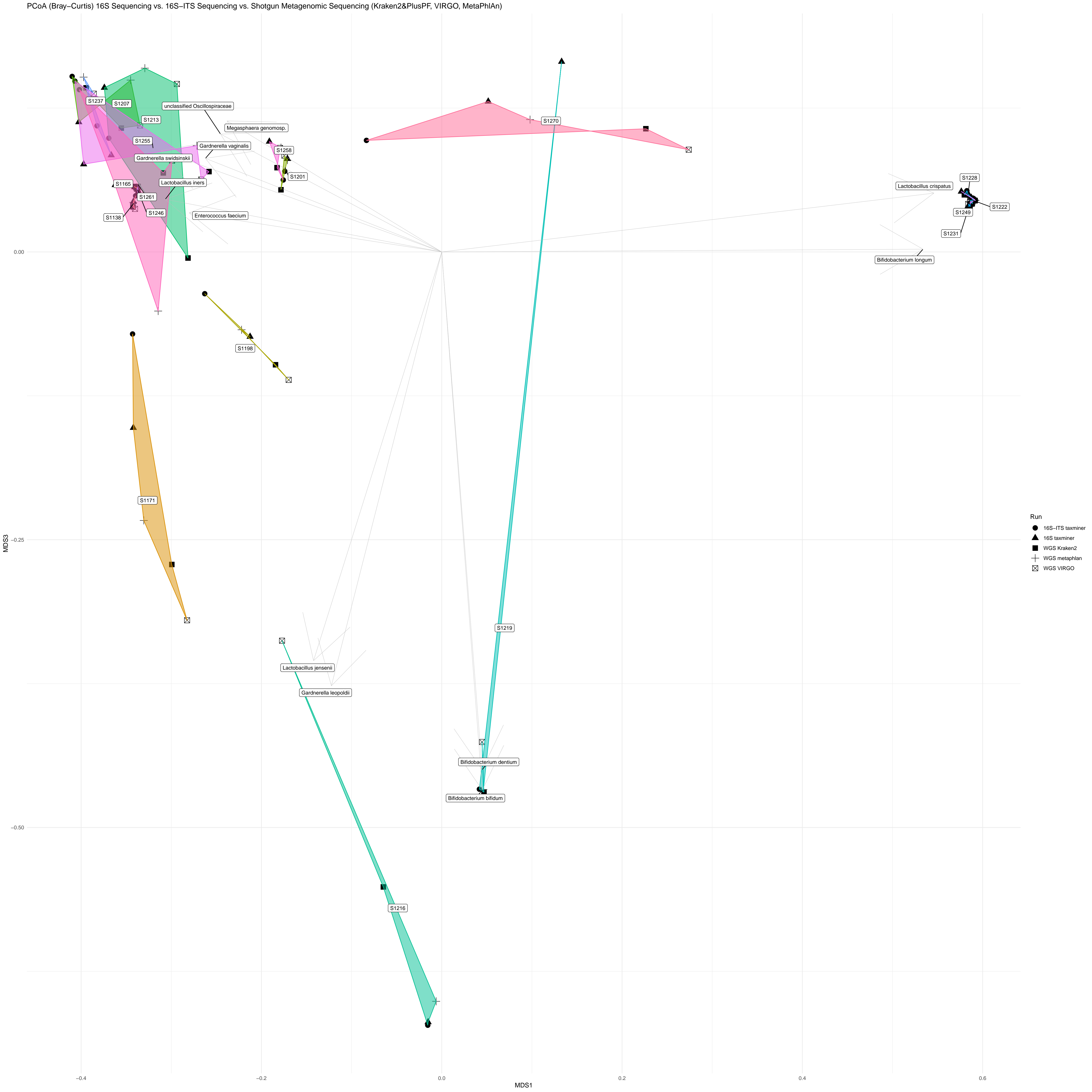

Supplement: Supplementary file 5 — Supplementary Material 4. Supplementary Figure S1: Principal coordinate analysis (PCoA) of the species level bacterial relative abundances. [file 40168_2024_1993_MOESM4_ESM.pdf]

# MiSeq\_ITS

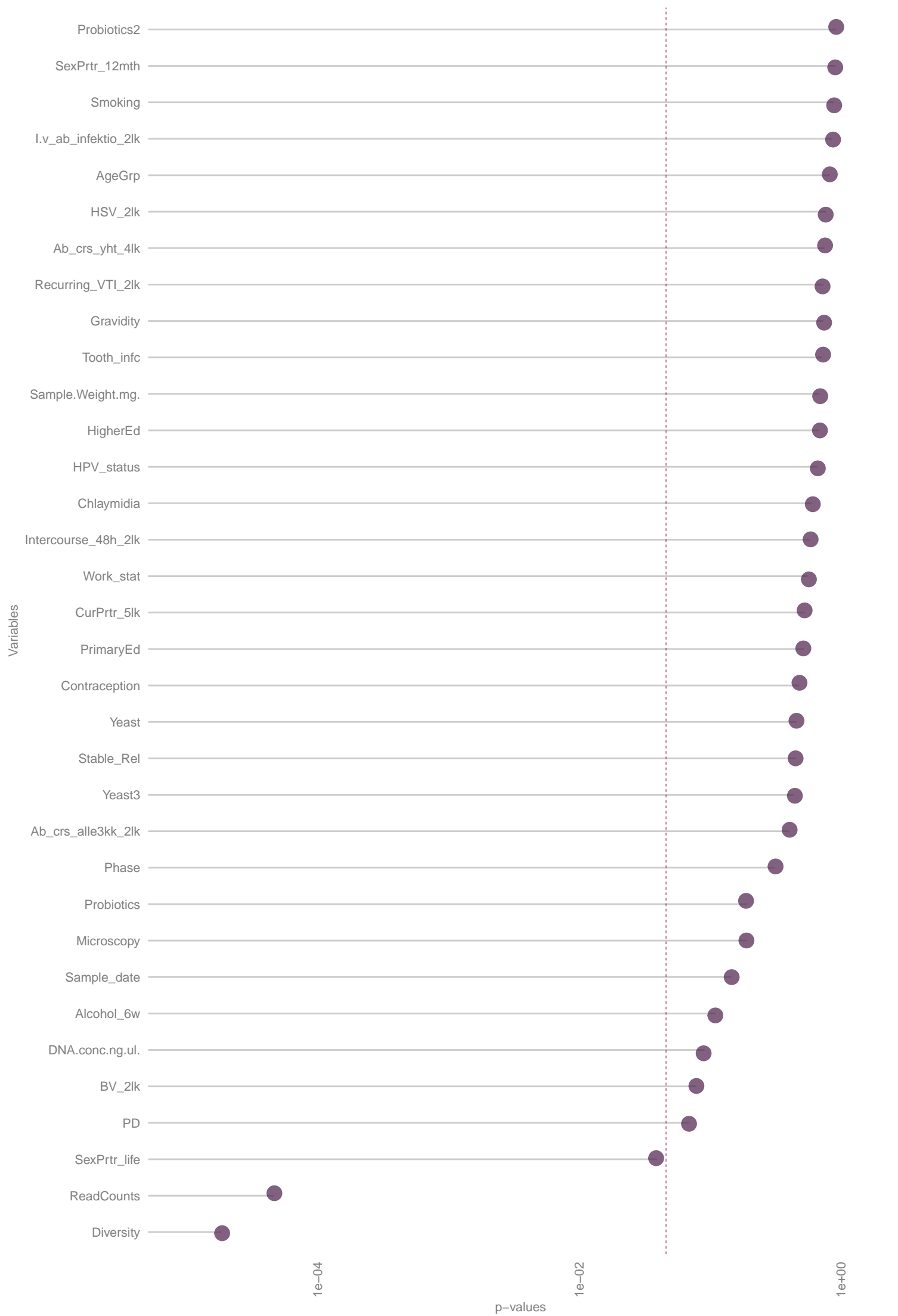

Supplement: Supplementary file 6 — Supplementary Material 5. Supplementary Figure S2: PERMANOVA results depicting the significance of background variables against the fungal profiles. [file 40168_2024_1993_MOESM5_ESM.pdf]

# Correlations (Pearson)

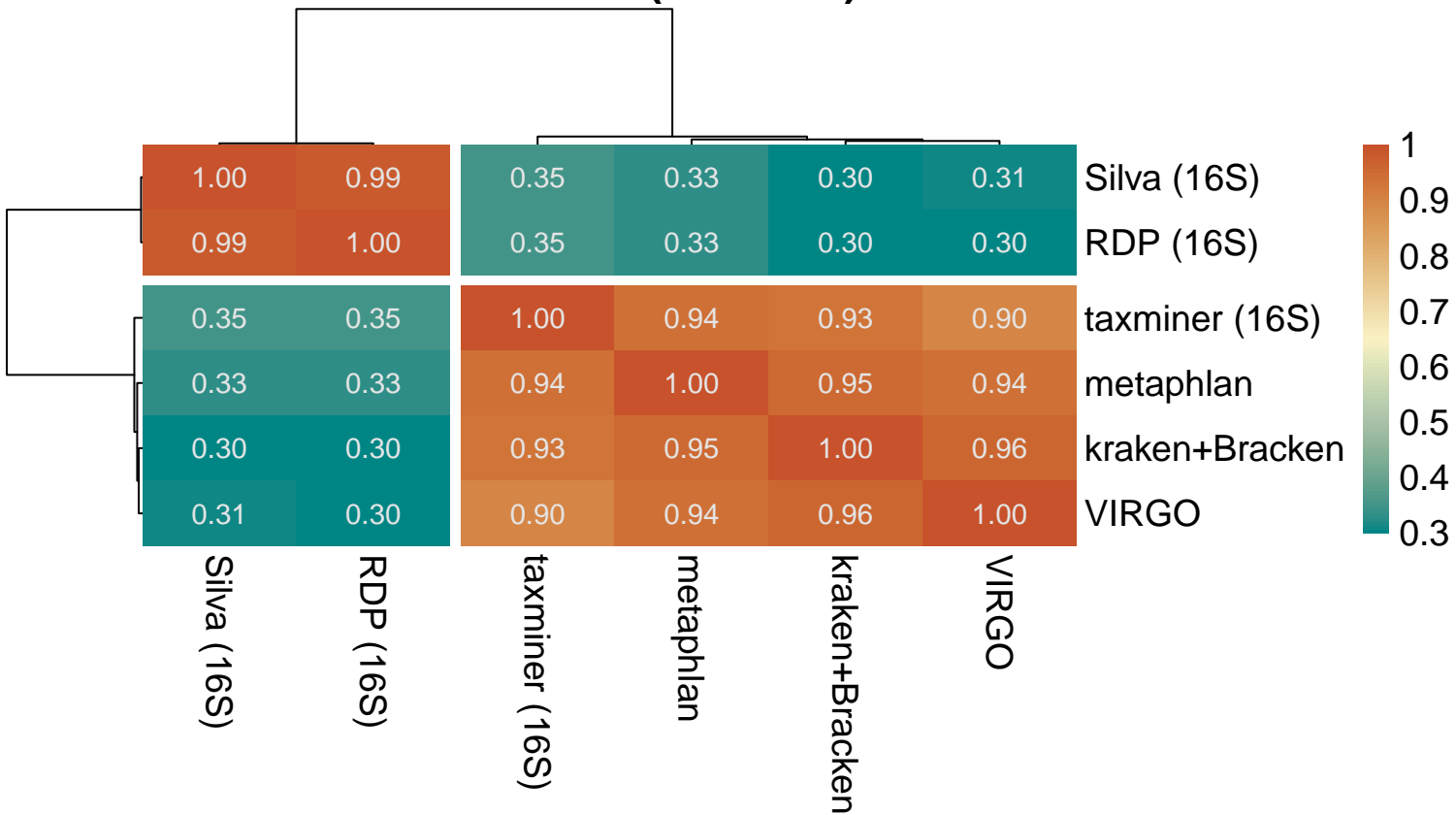

Supplement: Supplementary file 7 — Supplementary Material 6. Supplementary Figure S3: Pearsons correlations between the species level bacterial relative abundances obtained from the different taxonomic annotations tools and databases. [file 40168_2024_1993_MOESM6_ESM.pdf]

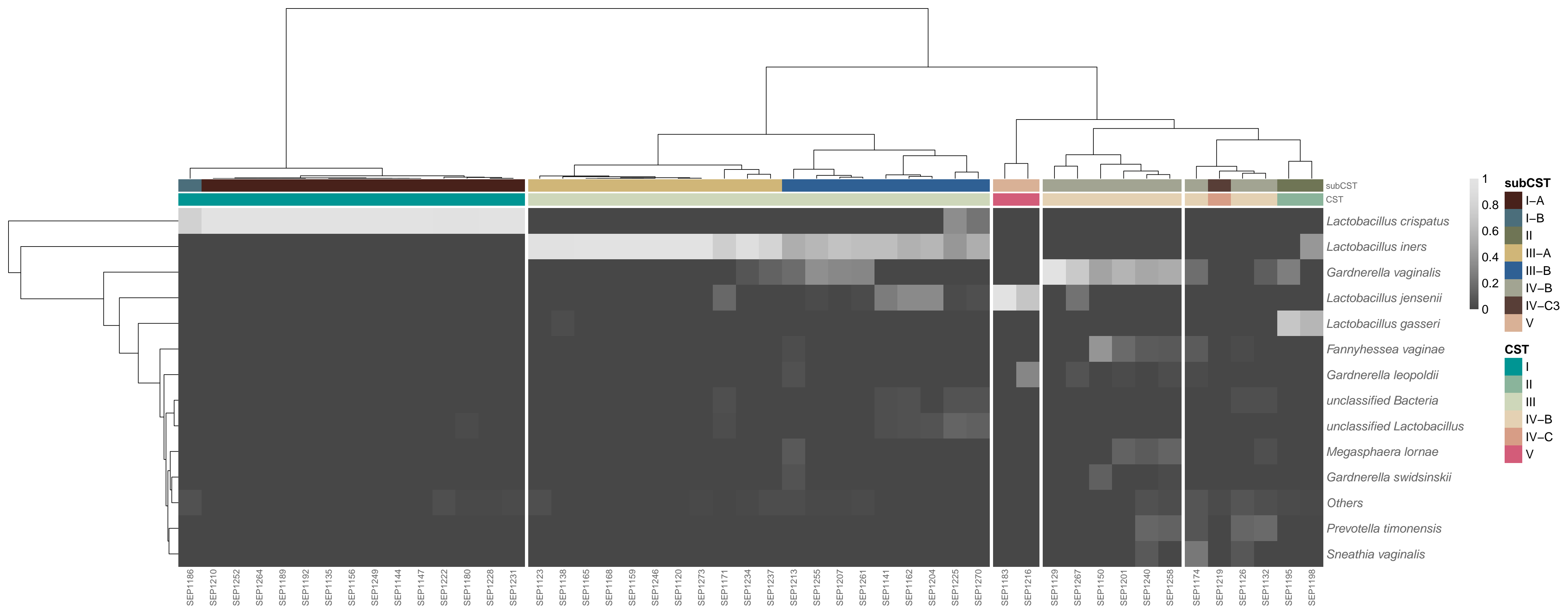

Supplement: Supplementary file 9 — Supplementary Material 8. Supplementary Figure S5: Community state types (CSTs) assigned to the bacterial profiles using the Valencia CST assignment tool. [file 40168_2024_1993_MOESM8_ESM.pdf]

Enzyme Abundance CPoM (Log transformed)

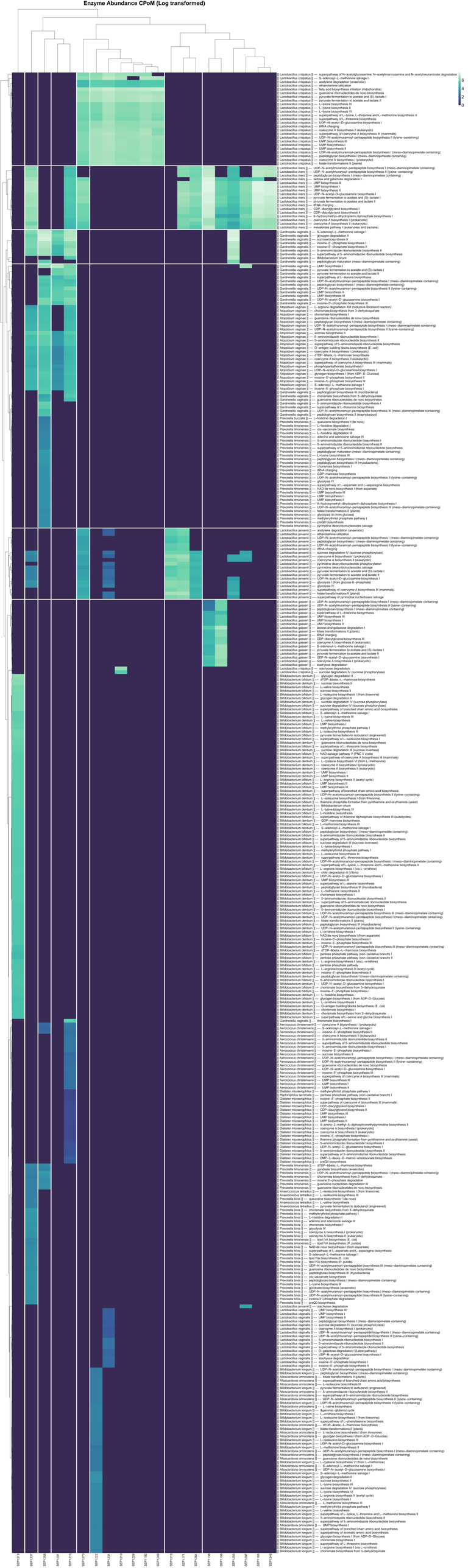

Supplement: Supplementary file 10 — Supplementary Material 9. Supplementary Figure S6: Heatmap illustrating the pathways identified in each sample through the Humann pipeline. [file 40168_2024_1993_MOESM9_ESM.pdf]
