# Supplementary material for: Metagenome-validated combined amplicon sequencing and text mining-based annotations for simultaneous profiling of bacteria and fungi: vaginal microbiota and mycobiota in healthy women
Source: Microbiome. 2024 Dec 28;12:273. doi: 10.1186/s40168-024-01993-9 (PMC11681650; doi:10.1186/s40168-024-01993-9)

FunOMIC fungal compositions for extremely deep metagenomic sequencing.
Zymomock contains equal amounts of *S. cerevisiae* and *C. neoformans.*

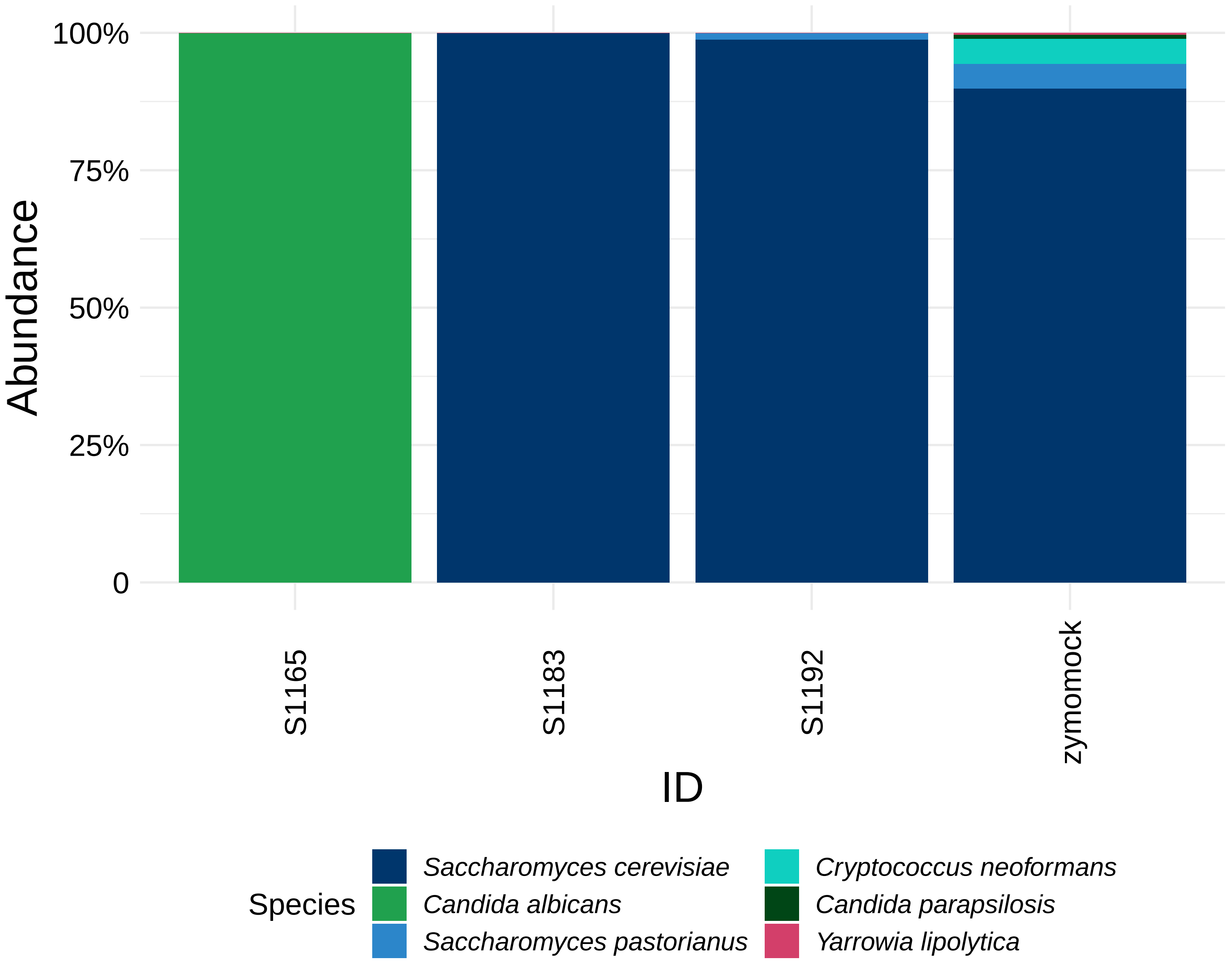

Supplement: Supplementary file 8 — Supplementary Material 7. Supplementary Figure S4: Fungal profiles obtained from the FunOMICs annotation tool. [file 40168_2024_1993_MOESM7_ESM.pdf]
