# Supplementary material for: Metagenome-validated combined amplicon sequencing and text mining-based annotations for simultaneous profiling of bacteria and fungi: vaginal microbiota and mycobiota in healthy women
Source: Microbiome. 2024 Dec 28;12:273. doi: 10.1186/s40168-024-01993-9 (PMC11681650; doi:10.1186/s40168-024-01993-9)
Supplement: Supplementary file 12 — Supplementary Material 11. Supplementary document 1: Results and discussion. [file 40168_2024_1993_MOESM11_ESM.docx]

Results: Functional profiles

The first cluster contained pathways that are essential to life and were observed across all samples. This included coenzyme A biosynthesis (COA-PWY), tRNA charging (TRNA-CHARGING-PWY), which consists of a group of 19 ligases responsible for amino acid activation, and cell wall biosynthesis pathways, mainly UDP-N-acetyl-D-glucosamine (UDPNAGSYN-PWY) and peptidoglycan biosynthesis (PEPTIDOGLYCANSYN-PWY). Pyruvate fermentation to lactate and acetate (PWY-5100) was largely observed in samples containing lactobacilli, highlighting their capacity to produce lactic acid.

A second cluster, representing pathways observed In samples containing L. crispatus (or CST I), consisted of amino acid biosynthesis, flavin biosynthesis, folate transformations, amino sugar degradation, and acetylene degradation pathways. There were nine enzymes related to the super pathway of L-lysine, L-threonine, and L-methionine biosynthesis (PWY-724), shared between L. crispatus and Bifidobacterium spp. but were absent in L. iners. A further 7 enzymes related to the L-lysine biosynthesis pathway (PWY-2941) were observed within this cluster, with pathway variant II, distinguished by the enzyme N-acetyl-diaminopimelate deacetylase (EC 3.5.1.47), exclusively seen in L. crispatus. Two other variants (III & VI) that share the remaining six enzymes were also observed in Bifidobacterium spp., while they were completely absent in L. iners. An amino sugar degradation pathway, super pathway of N-acetylglucosamine (GlcNAc), N-acetylmannosamine (ManNAc) and N-acetylneuraminate (NeuAc) degradation (GLCMANNANAUT-PWY), and acetylene degradation (P161-PWY) were also observed to be exclusive to L. crispatus.

Another group of sparsely distributed pathways was observed to be specific to samples containing L. iners (or CST III), which included CDP-diacylglycerol biosynthesis (PWY-5667), 6-hydroxymethyl-dihydropterin diphosphate biosynthesis (PWY-6147), and six enzymes from the mevalonate pathway (PWY-922). The last cluster of interest consisted of pathways observed only in samples containing Bifidobacterium spp. or G. vaginalis (CST IV C-3 and CST IV B). This included two pathways for the synthesis of inosine monophosphate (IMP); a super pathway of 5-aminoimidazole ribonucleotide biosynthesis (PWY-6277) and an inosine-’'-phosphate biosynthesis pathway (PWY-6123).

Discussion: Functional profiles

Even with a relatively small sample size, functional profiles obtained from the metagenome analysis revealed clear separation of bacterial profiles and their metabolic potential. The capacity for *L. crispatus* to synthesize lysine has been previously identified as well as its absence in *L. iners* [1]. An interesting observation was the co-occurrence of the almost complete mevalonate pathway (six enzymes), which produces cholesterol as an end-product, and inerolysin, a cholesterol dependent cytolysin (CDC) in samples containing *L. iners*. Presence of the mevalonate pathway has been indicated in urogenital bacterial populations and associated to the ability of certain lactic acid bacteria to cope with oxidative stress, which may be an additional adaptation that allows *L. iners* to colonize hostile environments as well as co-habit with other microbes, especially *G. vaginalis* [1–3]. CDCs such as inerolysin and vaginolysin (*G. vaginalis* specific) have been associated to membrane adhesion and subsequent pathogenicity. Pore formation in the host cell membranes, a characteristic of CDCs, has been speculated to allow the microbes to gain access to nutrients and potentially kill immune cells, allowing *L. iners* to survive in a dysbiotic state [1,4,5]. A repertoire of enzymes involved in the biosynthesis and degradation of inosine monophosphate (IMP) were seen in samples containing *G. vaginalis*. Even though the enzymes associated with degradation could not be assigned to a single taxonomy, their exclusivity in these samples already shows an interesting trend. Other studies have discussed inosine in various contexts, including as a metabolite from related intestinal *Bifidobacterium pseudolongum* that modulates an enhanced immunotherapy response, while for some strains of *G. vaginalis* it has been shown as a carbon source [6,7]. These findings warrant further investigations to understand the role of inosine metabolism among certain *G. vaginalis* strains within the vaginal ecosystem. The genus *Gardnerella* has recently been studied further to understand the strain level differences of *G. vaginalis* that may influence its capacity for pathogenicity [8]. Several new species have also been defined within the *Gardnerella* genus with varying characteristics that allow them to act as either a commensal or pathogenic microbe in the vaginal ecosystem [8]. *G. vaginalis* has been previously described to exhibit varying capacities for sialidase (an enzyme responsible for mucus degradation), and beta galactosidase production [9–12]. Different strains of *G. vaginalis* have been identified with varying degrees of virulence based on the presence/absence of the genetic machinery facilitating adherence, biofilm formation, cytotoxicity, glycan degradation, antibiotic resistance, and displacement of lactobacilli [11]. This is indicative of the tendency of some *G. vaginalis* strains to colonize a non-BV vaginal environment as a commensal microbe while other strains can exhibit pathogenicity, highlighting the importance of achieving strain level resolution to understand their biological characteristics and as a diagnostic tool.

1. France MT, Mendes-Soares H, Forney LJ. Genomic comparisons of Lactobacillus crispatus and Lactobacillus iners reveal potential ecological drivers of community composition in the vagina. Appl Environ Microbiol. 2016;82:7063–73.

2. Hagi T, Kobayashi M, Nomura M. Aerobic conditions increase isoprenoid biosynthesis pathway gene expression levels for carotenoid production in Enterococcus gilvus. FEMS Microbiol Lett. 2015;362:75.

3. Thomas-White K, Forster SC, Kumar N, Van Kuiken M, Putonti C, Stares MD, et al. Culturing of female bladder bacteria reveals an interconnected urogenital microbiota. Nat Commun. 2018;9:1–7.

4. Ragaliauskas T, Plečkaitytė M, Jankunec M, Labanauskas L, Baranauskiene L, Valincius G. Inerolysin and vaginolysin, the cytolysins implicated in vaginal dysbiosis, differently impair molecular integrity of phospholipid membranes. Sci Rep. 2019;9:1–11.

5. Rampersaud R, Planet PJ, Randis TM, Kulkarni R, Aguilar JL, Lehrer RI, et al. Inerolysin, a cholesterol-dependent cytolysin produced by Lactobacillus iners∇. J Bacteriol. 2011;193:1034–41.

6. Mager LF, Burkhard R, Pett N, Cooke NCA, Brown K, Ramay H, et al. Microbiome-derived inosine modulates response to checkpoint inhibitor immunotherapy. Science (1979). 2020;369:1481–9.

7. Khan S, Vancuren SJ, Hill JE. A Generalist Lifestyle Allows Rare Gardnerella spp. to Persist at Low Levels in the Vaginal Microbiome. Microb Ecol. 2020;1.

8. Vaneechoutte M, Guschin A, Van Simaey L, Gansemans Y, Van Nieuwerburgh F, Cools P. Emended description of Gardnerella vaginalis and description of gardnerella leopoldii sp. Nov., gardnerella piotii sp. nov. and Gardnerella swidsinskii sp. nov., with delineation of 13 genomic species within the genus Gardnerella. Int J Syst Evol Microbiol [Internet]. 2019 [cited 2021 Feb 9];69:679–87. Available from: https://www.microbiologyresearch.org/content/journal/ijsem/10.1099/ijsem.0.003200

9. Lewis WG, Robinson LS, Gilbert NM, Perry JC, Lewis AL. Degradation, foraging, and depletion of mucus sialoglycans by the vagina-adapted actinobacterium Gardnerella vaginalis. Journal of Biological Chemistry. 2013;288:12067–79.

10. Cornejo OE, Hickey RJ, Suzuki H, Forney LJ. Focusing the diversity of Gardnerella vaginalis through the lens of ecotypes. Evol Appl. 2018;11:312–24.

11. Castro J, Alves P, Sousa C, Cereija T, França Â, Jefferson KK, et al. Using an in-vitro biofilm model to assess the virulence potential of Bacterial Vaginosis or non-Bacterial Vaginosis Gardnerella vaginalis isolates. Sci Rep. 2015;5.

12. Vaneechoutte M, Guschin A, Van Simaey L, Gansemans Y, Van Nieuwerburgh F, Cools P. Emended description of Gardnerella vaginalis and description of gardnerella leopoldii sp. Nov., gardnerella piotii sp. nov. and Gardnerella swidsinskii sp. nov., with delineation of 13 genomic species within the genus Gardnerella. Int J Syst Evol Microbiol. 2019;69:679–87.
