# Supplementary material for: Metagenome-validated combined amplicon sequencing and text mining-based annotations for simultaneous profiling of bacteria and fungi: vaginal microbiota and mycobiota in healthy women
Source: Microbiome. 2024 Dec 28;12:273. doi: 10.1186/s40168-024-01993-9 (PMC11681650; doi:10.1186/s40168-024-01993-9)

## Enzyme Abundance Sp (Log transformed)

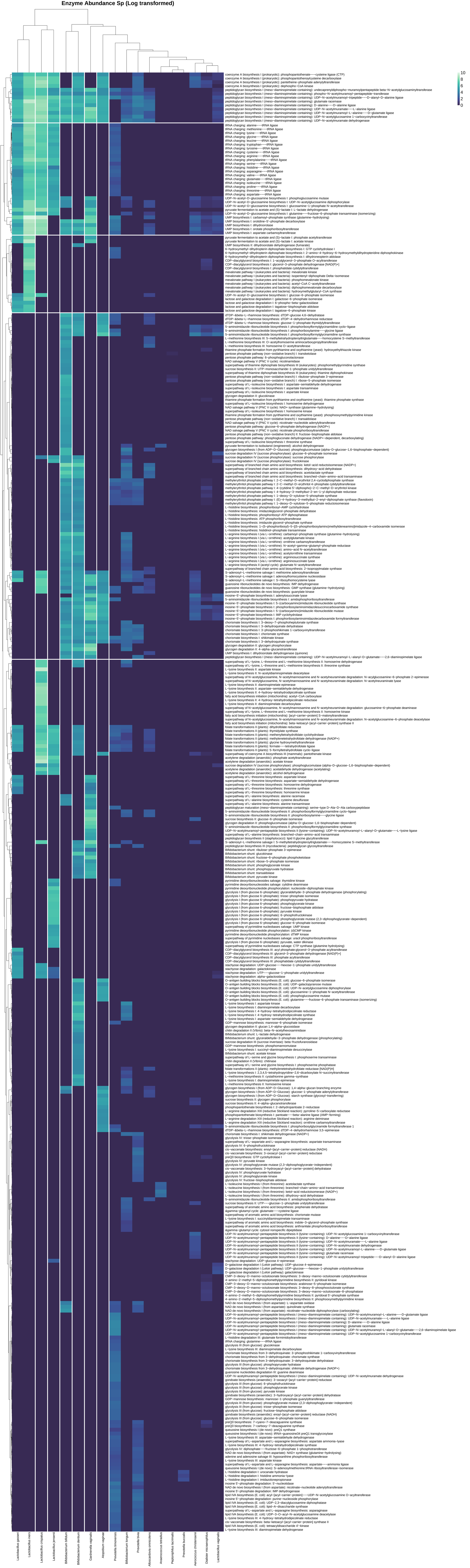

Supplement: Supplementary file 11 — Supplementary Material 10. Supplementary Figure S7: Heatmap illustrating the pathways identified against taxa through the Humann pipeline. [file 40168_2024_1993_MOESM10_ESM.pdf]
